# Unsupervised machine learning for identifying attention-deficit/hyperactivity disorder subtypes based on cognitive function and their implications for brain structure

**DOI:** 10.1017/S0033291724002368

**Published:** 2024-10

**Authors:** Masatoshi Yamashita, Qiulu Shou, Yoshifumi Mizuno

**Affiliations:** 1 Research Center for Child Mental Development, University of Fukui, Fukui, Japan; 2 Division of Developmental Higher Brain Functions, United Graduate School of Child Development, University of Fukui, Fukui, Japan; 3 Department of Child and Adolescent Psychological Medicine, University of Fukui Hospital, Fukui, Japan

**Keywords:** attention-deficit/hyperactivity disorder, brain structure, cognitive function, heterogeneity, unsupervised machine learning

## Abstract

**Background:**

Structural anomalies in the frontal lobe and basal ganglia have been reported in patients with attention-deficit/hyperactivity disorder (ADHD). However, these findings have been not always consistent because of ADHD diversity. This study aimed to identify ADHD subtypes based on cognitive function and find their distinct brain structural characteristics.

**Methods:**

Using the data of 656 children with ADHD from the Adolescent Brain Cognitive Development (ABCD) Study, we applied unsupervised machine learning to identify ADHD subtypes using the National Institutes of Health Toolbox Tasks. Moreover, we compared the regional brain volumes between each ADHD subtype and 6601 children without ADHD (non-ADHD).

**Results:**

Hierarchical cluster analysis automatically classified ADHD into three distinct subtypes: ADHD-A (*n* = 212, characterized by high-order cognitive ability), ADHD-B (*n* = 190, characterized by low cognitive control, processing speed, and episodic memory), and ADHD-C (*n* = 254, characterized by strikingly low cognitive control, working memory, episodic memory, and language ability). Structural analyses revealed that the ADHD-C type had significantly smaller volumes of the left inferior temporal gyrus and right lateral orbitofrontal cortex than the non-ADHD group, and the right lateral orbitofrontal cortex volume was positively correlated with language performance in the ADHD-C type. However, the volumes of the ADHD-A and ADHD-B types were not significantly different from those of the non-ADHD group.

**Conclusions:**

These results indicate the presence of anomalies in the lateral orbitofrontal cortex associated with language deficits in the ADHD-C type. Subtype specificity may explain previous inconsistencies in brain structural anomalies reported in ADHD.

## Introduction

Attention-deficit/hyperactivity disorder (ADHD) has a prevalence of > 5% in children and adolescents (Thomas, Sanders, Doust, Beller, & Glasziou, 2015). It is characterized by age-inappropriate symptoms of inattention, hyperactivity, and impulsivity (American Psychiatric Association, 2013). Moreover, ADHD is associated with an increased risk of psychiatric comorbidities such as depression and conduct disorders (Feldman & Reiff, 2014). Although the Diagnostic and Statistical Manual of Mental Disorders (DSM-5) provides diagnostic criteria for ADHD based on symptoms, significant neurobiological and cognitive heterogeneity remains. Psychoradiology applies medical imaging technologies to psychiatry and promises not only to improve insight into structural and functional brain abnormalities in patients with psychiatric disorders but also to have potential clinical utility (Luo, You, DelBello, Gong, & Li, 2022). Previous studies using magnetic resonance imaging (MRI) and cognitive function assessment have not always yielded consistent results, perhaps because of the heterogeneity, although they have tried to clarify the underlying ADHD pathology.

Previous studies revealed smaller gray matter volumes (GMVs) in the frontal lobe and basal ganglia in patients with ADHD than in healthy individuals (Bonath, Tegelbeckers, Wilke, Flechtner, & Krauel, 2018; Klein et al., 2021; Moreno-Alcázar et al., 2016; Villemonteix et al., 2015). Nonetheless, other studies found larger GMVs in the prefrontal-temporal-parietal areas in patients with ADHD than in healthy individuals (Seidman et al., 2011; Semrud-Clikeman, Pliszka, Bledsoe, & Lancaster, 2014; Wu et al., 2019). Moreover, larger ADHD studies on brain structure generally reported small effect sizes (Nakao, Radua, Rubia, & Mataix-Cols, 2011; Postema et al., 2021). This discrepancy between previous studies might reflect the diversity of the disorders themselves; ADHD is not a single pathology but an aggregate of heterogeneous pathologies.

Based on Barkley's theory, a growing body of evidence suggests various deficits in cognitive function, including executive function, in ADHD (Barkley, 1997). These studies have reported that patients with ADHD show impairments of response inhibition (Breitling-Ziegler, Tegelbeckers, Flechtner, & Krauel, 2020; Castellanos, Sonuga-Barke, Milham, & Tannock, 2006), non-verbal and verbal working memory (Anker, Ogrim, & Heir, 2022; Blomberg, Johansson Capusan, Signoret, Danielsson, & Rönnberg, 2021), processing speed (Castellanos et al., 2006; Mohamed et al., 2021), and speech production (Blomberg, Danielsson, Rudner, Söderlund, & Rönnberg, 2019). Although various executive function factors have been associated with ADHD, the evidence is not always consistent (Lacerda et al., 2020; Salari, Bohlin, Rydell, & Thorell, 2017; Willcutt, Doyle, Nigg, Faraone, & Pennington, 2005). One possible reason is the difference in executive function domains, such as updating, shifting, and inhibition (or common executive function), according to some models (Friedman et al., 2008; Miyake et al., 2000). Another possibility is the diversity of the executive function patterns. One study reported an association of inhibitory function with inattention and hyperactivity/impulsivity in ADHD (Cai et al., 2023); however, another study only observed such symptom association with working memory deficit (Salari et al., 2017). These findings suggest that ADHD results from underlying deficits in some executive function patterns, and such domains may help to clarify the diversity and pathogenesis of ADHD. Elucidating the neural structural system of cognitive function, including executive function, subtypes of ADHD may contribute to our understanding of inconsistent findings. However, investigations of the brain structure of ADHD subtypes have been limited to clinical symptom-related subtypes (Qureshi, Min, Jo, & Lee, 2016; Saad, Griffiths, & Korgaonkar, 2020; Serrallach, Groß, Christiner, Wildermuth, & Schneider, 2022), and little is known about ADHD subtypes classified by cognitive function domains.

This study aimed to clarify this gap in the literature by investigating whether ADHD subtypes based on cognitive function domains, including executive function, can have different effects on the brain structure of affected individuals. The present findings may shed new light on ADHD heterogeneity and its pathogenesis. A better understanding of such heterogeneity in ADHD may be helpful for considering outcomes/prognoses or treatment planning.

## Methods

### Participants

The Adolescent Brain Cognitive Development (ABCD) Study is the largest longitudinal study examining child brain development and mental health in the United States (Jernigan, Brown, & ABCD Consortium Coordinators, 2018). Recruitment began in 2016 and ended in 2018; however, the study is ongoing and actively collecting longitudinal data. Full recruitment details of the ABCD Study have been published previously (Garavan et al., 2018). Briefly, the ABCD Study primarily recruited participants through elementary schools, both public (including charter schools) and private (Garavan et al., 2018). The dataset consisted of 11 878 adolescents aged 9 to 11 years (mean age, 9.91 years [min, 8.91; max, 11.08]) recruited from 21 data collection sites, namely Oregon Health and Science University, Stanford Research Institute, Children's Hospital Los Angeles, University of California at Los Angeles, University of California San Diego, University of Utah, University of Colorado Boulder, Laureate Institute for Brain Research, University of Minnesota, Washington University, University of Florida, Florida International University, University of Wisconsin-Milwaukee, University of Michigan, Medical University of South Carolina, University of Pittsburgh, Virginia Commonwealth University, University of Maryland at Baltimore, University of Rochester, Yale University, and University of Vermont, as previously reported (Cheng et al., 2021; Hiraoka, Makita, Hamatani, Tomoda, & Mizuno, 2023; Wiker et al., 2023; Zhang et al., 2022a). The present study mainly used data from the ABCD 2.0 release. All parents provided written informed consents, and all children provided assent. All procedures complied with the Declaration of Helsinki. The Research Ethics Committee of the University of Fukui approved the data analysis (Assurance No. FU-20210067).

The variables used in this study are shown in online Supplementary Table S1, and we used the baseline data, e.g., the initial data collected at the beginning of the ABCD Study, for analysis. First, the current ADHD/non-ADHD diagnoses were based on the computerized Kiddie Schedule for Affective Disorders and Schizophrenia (K-SADS) from the parents. The current episode diagnoses were based on computer self-administered K-SADS which shows high concordance rates (percent agreement in diagnostic categories: 88–96%) with the clinician-administered paper-and-pencil version of the K-SADS (Kobak, Kratochvil, Stanger, & Kaufman, 2013). The diagnoses were extracted using the identifier marked with ‘1’ as an ADHD diagnosis and marked with ‘0’ as a non-ADHD diagnosis, as previously reported (Bernanke et al., 2022; Kim et al., 2023). A total of 3372 participants with no data on diagnosis at baseline were excluded. Second, quality control of structural imaging data and FreeSurfer cortical surface reconstructions were performed manually by the ABCD team, and 188 participants who had no T1 quality checks or imaging data were excluded. Third, duplicate participants caused by the binding of all data tables were removed (*n* = 501). After the primary data-cleaning process (*n* = 7817), missing values were excluded from the current ADHD/non-ADHD diagnoses (*n* = 106). As reported in previous studies (Ma et al., 2022; Zhao et al., 2022), we excluded missing values from the National Institutes of Health (NIH) Toolbox Tasks (*n* = 147) because the imputation approach may have caused bias, such as cluster bias, in the results (van Rossum, da Silva, Wang, Kouwenhoven, & Hermens, 2023). Moreover, we excluded cases ineligible for the T1 quality check (*n* = 307), which were extracted using the identifier marked with ‘0’ as unacceptable imaging results by the ABCD team. The final analysis included 7257 participants (see online Supplementary Fig. S1 for a flowchart of the sampling procedure). Statistical analyses, including data cleaning (R package ‘dplyr’), were conducted using R (version 4.3.0; The R Foundation for Statistical Computing, Vienna, Austria).

### Demographic variables and covariates

The following covariates were included as categorical variables and dummy-coded: sex, handedness, race/ethnicity (White, Black, Hispanic, Asian, and other), and medication use. Based on previous studies (Hamatani, Hiraoka, Makita, Tomoda, & Mizuno, 2022; Hiraoka et al., 2023; Paul et al., 2021), annual household income was treated as a five-level categorical variable (Table 1). The following covariates were included as continuous variables: age, parental education level, pubertal status, and total intracranial volume. Parental educational level was recorded as follows: 12th grade, high school, and general education, 12 years; college and associate degrees, 14 years; bachelor's degree, 16 years; master's degree, 18 years; and professional and doctoral degrees, 20 years. The pubertal development scale was used to assess pubertal status (Petersen, Crockett, Richards, & Boxer, 1988). It was completed by both a parent or guardian and the participant, and the results of the two scores were averaged. The abovementioned covariates were selected based on previous ABCD-based studies (Bernanke et al., 2022; Hamatani et al., 2022; Hiraoka et al., 2023; Owens et al., 2021).

**Table 1.** Baseline demographics of the ADHD and non-ADHD groups

Characteristic	Non-ADHD (*n* = 6601)	ADHD (*n* = 656)	*p* Value
Age (months)	119.04 (7.48)	118.74 (7.53)	0.328
Parental education (years)	15.22 (2.62)	15.37 (2.43)	0.181
Pubertal status (score)	1.61 (0.50)	1.56 (0.50)	0.018
Sex (*n*)			
Male	3328 (50.42)	451 (68.75)	< 0.001
Female	3273 (49.58)	205 (31.25)	
Race/ethnicity (*n*)			
White	3477 (52.67)	352 (53.66)	0.662
Black	957 (14.50)	110 (16.77)	0.132
Hispanic	1345 (20.37)	98 (14.94)	0.001
Asian	144 (2.18)	5 (0.76)	0.021
Other	677 (10.26)	91 (13.87)	0.005
Annual household income (US$) (*n*)			
< 49 999	1735 (26.28)	189 (28.81)	0.090
50 000–74 999	822 (12.45)	85 (12.96)	
75 000–99 999	917 (13.89)	96 (14.63)	
100 000–199 999	1907 (28.89)	167 (25.46)	
⩾ 200 000	680 (10.30)	66 (10.06)	
CBCL (score)			
ADHD symptom	52.10 (4.10)	63.43 (7.61)	< 0.001
Depressive symptom	52.95 (4.99)	59.18 (7.87)	< 0.001
Symptom of anxiety disorder	52.96 (5.50)	58.73 (9.48)	< 0.001
Symptom of conduct disorder	52.36 (4.67)	58.09 (8.34)	< 0.001
Symptom of oppositional defiant disorder	52.75 (4.42)	59.38 (8.47)	< 0.001
Symptom of somatic disorder	55.25 (6.41)	57.97 (7.53)	< 0.001

ADHD, attention-deficit/hyperactivity disorder; CBCL, Child Behavior Checklist; s.d., standard deviation.

Data are presented as the mean (s.d.) or *n* (%). *p* values for age, education, puberty, income, and CBCL scores are from *t* tests for group differences. *p* values for sex ratio and race/ethnicity ratio are from χ^2^ tests for group differences.

For additional demographic clinical variables, ADHD symptoms and psychiatric onset risk were assessed using the six DSM-5-oriented syndrome scales (symptoms of ADHD, depression, anxiety disorder, conduct disorder, oppositional defiant disorder, and somatic disorder) of the Child Behavior Checklist (CBCL) (Achenbach & Rescorla, 2001). We used *T* scores calculated by the ABCD team, with higher scores representing greater behavioral problems.

### Behavioral measurements and statistical analysis

NIH Toolbox Tasks, measured using an iPad-based program, were used to classify ADHD subtypes based on cognitive function domains, including executive function. The cognition battery comprised seven measures to assess five domains: executive function and attention (also known as cognitive control; using flanker inhibitory control and attention and dimensional change card sort), processing speed (using pattern comparison processing speed), working memory (using list sorting working memory), episodic memory (using picture sequence memory), and language (using picture vocabulary and oral reading recognition) (Fox, Manly, Slotkin, Devin Peipert, & Gershon, 2021; Nolin et al., 2023; Ott et al., 2022; see online Supplementary Methods S1 for the details of each task). Age-corrected scores were used, and the composite score of cognitive control was created by adding the flanker inhibitory control and attention and dimensional change card sort scores, whereas the composite score of language was created by adding the picture vocabulary and oral reading recognition scores, according to previous studies on the classification of the cognitive function domains of the NIH Toolbox (Nolin et al., 2023; Ott et al., 2022). Outliers were Winsorised at three standard deviations from the mean (R package ‘DescTools’).

Behavioral data were analyzed using unsupervised machine learning to classify the ADHD subtypes. This is based on previous studies that applied unsupervised machine learning to classify patient subtypes based on molecular and brain functions (Williams et al., 2023; Yu, Wang, Ge, & Shi, 2022; Zhang, Manza, & Volkow, 2022b). Moreover, cluster analysis in this study was restricted to participants with ADHD as well as methods in studies (Scheerer et al., 2021; Yu et al., 2022). Thereafter, data were compared between each identified ADHD subtype and the non-ADHD group. To mitigate distance-associated issues in the cluster analysis, the behavioral scores were converted to *z* scores and hierarchically clustered to identify patterns of cognitive domains in this ADHD sample. The similarity between observations was calculated as the Euclidean distance and then clustered into distinct shapes followed by clustering based on magnitude using Ward's D2 method (R package ‘NbClust’). Based on the Ball-Hall index (Ball & Hall, 1965), the number of clusters was automatically determined by the system. The support vector machine (SVM; R package ‘e1071’) supporting multi-class classifiers with one-*v.*-one approach was used to verify the reliability and reproducibility of the subtyping based on cognitive function domains. This simulation learning was repeated 1000 times for each training dataset (75% of the 656 participants with ADHD, *n* = 492) and test dataset (25% of the 656 participants with ADHD, *n* = 164). The uniform manifold approximation and projection (UMAP; R packages ‘umap’ and ‘ggplot2’) algorithm applying the Euclidean distance was used to visualize the cluster plot.

To assess cognitive features by comparing each ADHD subtype with the non-ADHD group, a linear mixed-effects model (R packages ‘lmerTest’, ‘MuMIn’, and ‘jtools’) was used with each cognitive function as the dependent variable and group (e.g. ADHD-A *v.* non-ADHD) as the independent variable. Based on previous studies (Hamatani et al., 2022; Hiraoka et al., 2023), family ID (used to denote sibling status), multiple data collection sites, and twin or triplet status were modeled as random effects. Covariates included the abovementioned variables. The statistical threshold was set at *p* < 0.05, false discovery rate (FDR)-corrected using the Benjamini–Hochberg method. Thereafter, corrections for the family-wise error (FWE, *p* < 0.05) rate were performed using the Bonferroni method for multiple group comparisons. For additional analyses using the linear mixed-effects model adjusted for comorbidities, see online Supplementary Methods S2.

### Structural data and statistical analysis

Scanning was performed using three 3 T MR scanners (Siemens, General Electric 750, and Philips) to obtain high-resolution T1-weighted three-dimensional structural images (1 mm isotropic) with acquisition parameters as described previously (Casey et al., 2018). Structural data were preprocessed by the ABCD data team using the standard morphometric pipeline (e.g. skull-stripping, white matter segmentation) in FreeSurfer software (version 5.3.0) (Hagler et al., 2019). We used 34 regions labeled with the Desikan atlas-based classification for cortical regional volume and six regions labeled with atlas-based segmentation for subcortical regional volume (68 and 12 regions in total, respectively). Outliers were Winsorised at three standard deviations from the mean.

To assess brain structural characteristics by comparing each ADHD subtype with the non-ADHD group, a linear mixed-effects model was used with each regional brain volume as the dependent variable and group (e.g. ADHD-A *v.* non-ADHD) as the independent variable. In addition to multiple data collection sites and twin or triplet status, we included the family ID as a random effect nested inside a random effect of the MRI scanner to account for the large number of siblings and multiple data collection sites, as recommended by previous studies (Bernanke et al., 2022; Hagler et al., 2019; Heeringa & Berglund, 2020; Owens et al., 2021). The reason is the characteristics of the protocols in the ABCD Study: the data collection includes the recruitment of multiple students from schools, multiple children recruited from the same family, multiple children imaged on the same MRI scanner, and the use of multiple scanners at the same site (Heeringa & Berglund, 2020). Moreover, covariates included the abovementioned variables and total intracranial volume. The statistical threshold was set at *p* < 0.05, FDR-corrected using the Benjamini–Hochberg method. Thereafter, corrections for the FWE (*p* < 0.05) rate were performed using the Bonferroni method for multiple group comparisons. For additional analyses using the linear mixed-effects model adjusted for comorbidities, see online Supplementary Methods S2. Furthermore, based on differences in brain structural and cognitive functional results between groups (e.g. ADHD-C *v.* non-ADHD), we investigated associations between region-of-interest volumes and specific cognitive domains, using Pearson's correlation analyses (R package ‘psych’). The statistical threshold was set at *p* < 0.05, FDR-corrected using the Benjamini–Hochberg method.

## Results

### Demographics and cognitive function pattern clustering

**Figure 1.**
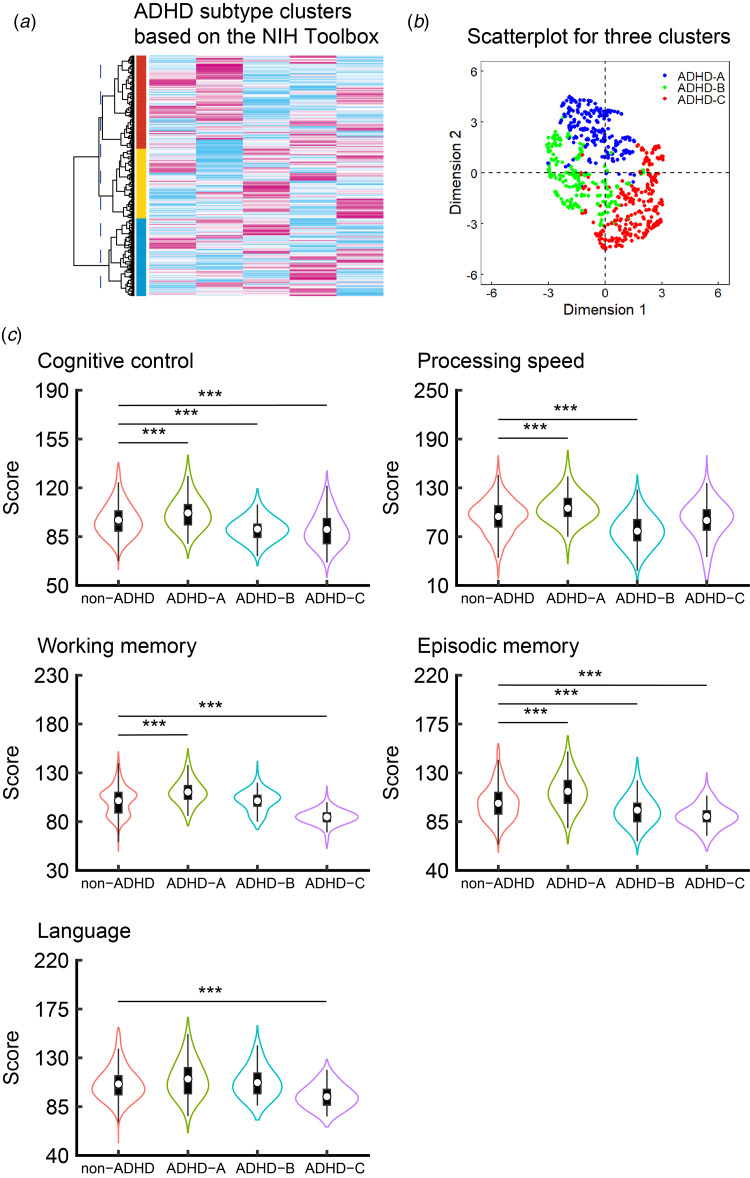
Cognitive function characteristics of ADHD subtypes based on the National Institutes of Health Toolbox Tasks. (a) Hierarchical cluster analysis identified three clusters based on NIH Toolbox Tasks. (b) Scatterplot performed by UMAP applying the Euclidean distance to visualize the three clusters. (c) Based on FDR-corrected and FWE-corrected thresholds (*p* < 0.05), the ADHD-A type showed better cognitive control, processing speed, working memory, and episodic memory than the non-ADHD group. The ADHD-B type showed poorer performances in cognitive control, processing speed, and episodic memory than the non-ADHD group. The ADHD-C type showed poorer performances in cognitive control, working memory, episodic memory, and language ability than the non-ADHD group. Parameters are indicated as the mean (s.d.). *** FDR-*p* < 0.001. ADHD, attention-deficit/hyperactivity disorder; FDR, false discovery rate; FWE, family-wise error; *SD*, standard deviation; UMAP, uniform manifold approximation and projection.

Baseline demographic data of 7257 children (6601 non-ADHD and 656 with ADHD) were obtained (Table 1). Hierarchical cluster analysis identified three clusters based on the five cognitive domains (Fig. 1a). A scatterplot of the three clusters obtained using the UMAP algorithm is shown in Fig. 1b. The three subtypes were named ADHD-A (*n* = 212), ADHD-B (*n* = 190), and ADHD-C (*n* = 254). In subsequent SVM algorism, the result showed high prediction accuracy and recall in the test dataset (accuracy, 0.93 ± 0.02; precision, 0.92 ± 0.04; recall, 0.88 ± 0.05; F1 score, 0.90 ± 0.03), suggesting that the cognitive function model may accurately identify the true positive class. The demographic characteristics of each subtype are shown in online Supplementary Result 1 and Supplementary Table S2.

### Cognitive features

As shown in Fig. 1c and Table 2, the main effect of group in the linear mixed-effects model showed that the ADHD-A type had significantly higher levels of cognitive control, processing speed, working memory, and episodic memory (FDR, *p* < 0.001 for each variable and FWE, *p* < 0.001 for each variable) than the non-ADHD group. Moreover, the main effect of group showed that the ADHD-B type had significantly lower levels of cognitive control, processing speed, and episodic memory (FDR, *p* < 0.001 for each variable and FWE, *p* < 0.001 for each variable) than the non-ADHD group. Furthermore, the main effect of group showed that the ADHD-C type had lower levels of cognitive control, working memory, episodic memory, and language (FDR, *p* < 0.001 for each variable and FWE, *p* < 0.001 for each variable) than the non-ADHD group. For significance comparing each ADHD subtype and the non-ADHD group adjusted for comorbidities, see online Supplementary Results 2, Supplementary Table S3, and Supplementary Fig. S2.

**Table 2.** Differences in behavioral results between each ADHD subtype and the non-ADHD group

Variable	*b*	95% CI	*β*	95% CI	*R*^2^	*t*	*F*	*d.f.*	FDR-*p*	FWE-*p*
ADHD-A										
Cognitive control	4.14	2.38 to 5.90	0.06	0.03 to 0.08	0.06	4.61	21.23	5834.92	< 0.001	< 0.001
Processing speed	9.43	6.23 to 12.63	0.08	0.05 to 0.10	0.04	5.77	33.29	5832.03	< 0.001	< 0.001
Working memory	6.90	4.86 to 8.94	0.08	0.06 to 0.11	0.12	6.63	44.01	5838.39	< 0.001	< 0.001
Episodic memory	9.95	7.63 to 12.26	0.11	0.08 to 0.13	0.08	8.44	71.17	5839.13	< 0.001	< 0.001
Language	1.66	−0.37 to 3.69	0.02	−0.004 to 0.04	0.21	1.60	2.56	5769.46	0.109	0.327
ADHD-B										
Cognitive control	−3.13	−4.03 to −2.23	−0.09	−0.11 to −0.06	0.06	6.81	46.44	5820.20	< 0.001	< 0.001
Processing speed	−8.30	−9.98 to −6.69	−0.13	−0.15 to −0.10	0.05	9.92	98.40	5801.16	< 0.001	< 0.001
Working memory	−0.41	−1.46 to 0.63	−0.01	−0.03 to 0.01	0.10	0.77	0.60	5801.40	0.712	> 0.999
Episodic memory	−2.78	−3.97 to −1.60	−0.06	−0.08 to −0.03	0.07	4.62	21.37	5792.22	< 0.001	< 0.001
Language	−0.19	−1.23 to 0.84	−0.004	−0.03 to 0.02	0.21	0.37	0.14	5545.54	0.549	> 0.999
ADHD-C										
Cognitive control	−1.79	−2.35 to −1.23	−0.08	−0.11 to −0.06	0.06	6.23	38.84	5853.75	< 0.001	< 0.001
Processing speed	−0.89	−1.92 to 0.14	−0.02	−0.05 to 0.004	0.03	1.69	2.86	5851.01	0.091	0.273
Working memory	−5.03	−5.67 to −4.38	−0.20	−0.22 to −0.17	0.14	15.2	232.31	5858.09	< 0.001	< 0.001
Episodic memory	−3.16	−3.90 to −2.43	−0.11	−0.14 to −0.09	0.08	8.47	71.76	5859.04	< 0.001	< 0.001
Language	−3.13	−3.77 to −2.49	−0.12	−0.14 to −0.09	0.23	9.56	91.42	5814.03	< 0.001	< 0.001

ADHD, attention-deficit/hyperactivity disorder; *b*, unstandardized coefficient; *β*, standardized coefficient; CI, confidence interval; *d.f*., degree of freedom; FDR, false discovery rate; FWE, family-wise error.

### Structural changes

**Figure 2.**
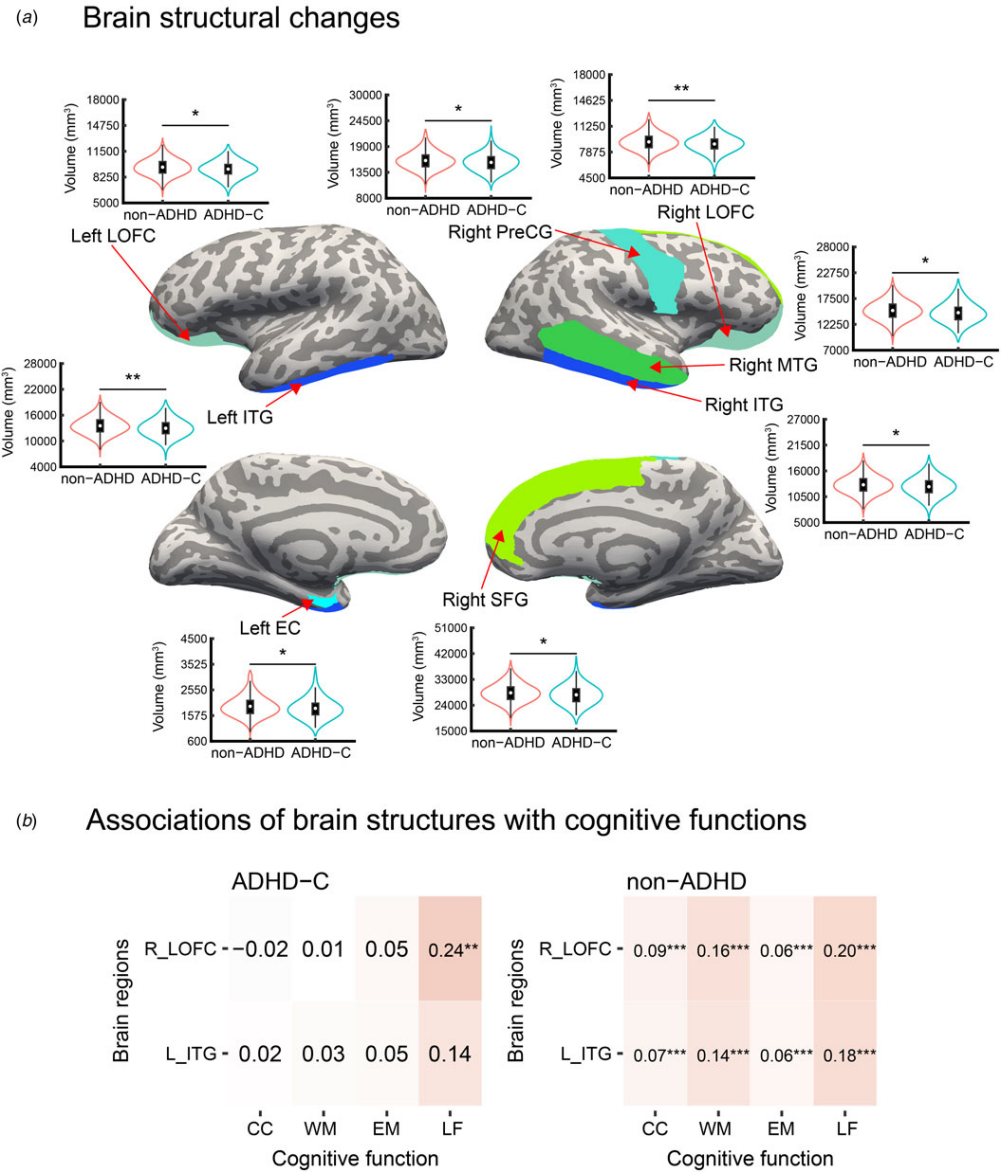
(a) Brain regions in which the ADHD-C type showed smaller volumes than the non-ADHD group and (b) Associations of brain structures (left inferior temporal gyrus and right lateral orbitofrontal cortex) with cognitive functions in the ADHD-C and non-ADHD groups. Based on FDR-corrected and FWE-corrected thresholds (*p* < 0.05), the ADHD-C type showed lower volumes in the left inferior temporal gyrus and right lateral orbitofrontal cortex than the non-ADHD group. Based on the FDR-corrected threshold (*p* < 0.05), the ADHD-C type showed lower volumes in the left entorhinal cortex, left lateral orbitofrontal cortex, right middle temporal gyrus, right precentral gyrus, right superior frontal gyrus, and right inferior temporal gyrus than the non-ADHD group. Only associations based on FWE-corrected thresholds were considered, where the ADHD-C group had smaller volumes and lower cognitive performances than the non-ADHD group. The ADHD-C group showed a positive correlation between volumes in the right lateral orbitofrontal cortex and language performances. In the non-ADHD group, both regional volumes were positively correlated with cognitive control, working memory, episodic memory, and language performances. In Fig. 2a, parameters are indicated as the mean (s.d.). In Fig. 2b, parameters are indicated as the *r* value. * FDR-*p* < 0.05, ** FDR-*p* < 0.01, *** FDR-*p* < 0.001. ADHD, attention-deficit/hyperactivity disorder; CC, cognitive control; EC, entorhinal cortex; EM, episodic memory; FDR, false discovery rate; FWE, family-wise error; ITG, inferior temporal gyrus; L_ITG, left inferior temporal gyrus; LF, language function; LOFC, lateral orbitofrontal cortex; MTG, middle temporal gyrus; PreCG, precentral gyrus; R_LOFC, right lateral orbitofrontal cortex; *SD*, standard deviation; SFG, superior frontal gyrus; WM, working memory.

As shown in Fig. 2a and Table 3, the main effect of group in the linear mixed-effects model showed that the ADHD-C type exhibited smaller volumes in the left inferior temporal gyrus (FDR, *p* = 0.002; FWE, *p* = 0.006) and right lateral orbitofrontal cortex (FDR, *p* = 0.004; FWE, *p* = 0.012) than the non-ADHD group. As for weak results, the main effect of group showed that the ADHD-C type had smaller volumes in the left entorhinal cortex (FDR, *p* = 0.046; FWE, *p* = 0.138), left lateral orbitofrontal cortex (FDR, *p* = 0.037; FWE, *p* = 0.111), right middle temporal gyrus (FDR, *p* = 0.036; FWE, *p* = 0.108), right precentral gyrus (FDR, *p* = 0.049; FWE, *p* = 0.147), right superior frontal gyrus (FDR, *p* = 0.037; FWE, *p* = 0.111), and right inferior temporal gyrus (FDR, *p* = 0.049; FWE, *p* = 0.147) than the non-ADHD group. However, the ADHD-A and ADHD-B types did not show such significant differences in regional brain volumes. For significance comparing each ADHD subtype and the non-ADHD group adjusted for comorbidities, see online Supplementary Results 3, Supplementary Table S4, and Supplementary Fig. S3.

**Table 3.** Brain areas with significantly smaller volume in the ADHD-C type than in the non-ADHD group

Brain region	*b*	95% CI	*β*	95% CI	*R*^2^	*t*	*F*	*d.f.*	FDR-*p*	FWE-*p*
Left										
Entorhinal	−24.13	−40.28 to −7.98	−0.04	−0.06 to −0.01	0.21	2.93	8.58	5744.09	0.046	0.138
Lateral orbitofrontal	−51.54	−86.89 to −16.18	−0.03	−0.05 to −0.01	0.51	2.86	8.16	5732.76	0.049	0.147
Inferior temporal	−159.08	−233.83 to −84.33	−0.04	−0.06 to −0.02	0.42	4.17	17.40	5741.40	0.002	0.006
Right										
Middle temporal	−107.36	−172.94 to −41.79	−0.03	−0.05 to −0.01	0.52	3.21	10.30	5740.49	0.036	0.108
Precentral	−96.74	−164.03 to −29.44	−0.03	−0.05 to −0.01	0.47	2.82	7.94	5743.47	0.049	0.147
Superior frontal	−164.89	−270.91 to −58.88	−0.03	−0.05 to −0.01	0.51	3.05	9.29	5740.61	0.037	0.111
Lateral orbitofrontal	−73.60	−110.61 to −36.59	−0.04	−0.06 to −0.02	0.38	3.90	15.19	5737.35	0.004	0.012
Inferior temporal	−112.98	−184.99 to −40.96	−0.03	−0.05 to −0.01	0.43	3.07	9.45	5738.63	0.037	0.111

ADHD, attention-deficit/hyperactivity disorder; *b*, unstandardized coefficient; *β*, standardized coefficient; CI, confidence interval; *d.f*., degree of freedom; FDR, false discovery rate; FWE, family-wise error.

Subsequently, we investigated correlations between the volumes of the left inferior temporal gyrus and right lateral orbitofrontal cortex and the scores of cognitive control, working memory, episodic memory, and language because these areas and cognitive functions significantly differed between the ADHD-C type and non-ADHD groups (Fig. 2b). In the ADHD-C type, the volume of the right lateral orbitofrontal cortex was positively correlated with language score (FDR, *p* = 0.005). In the non-ADHD group, the volumes of the left inferior temporal gyrus and right orbitofrontal cortex were both positively correlated with the scores of cognitive control, working memory, episodic memory, and language (FDR, *p*s < 0.001).

## Discussion

This study aimed to identify ADHD subtypes based on cognitive function domains, including executive function, using unsupervised clustering algorithms and to find distinct structural characteristics in the neural system of these subtypes. Clustering revealed three distinct ADHD subtypes (Fig. 1): the ADHD-A type was characterized by high cognitive function scores, the ADHD-B type was characterized by low cognitive control, processing speed, and episodic memory scores, and the ADHD-C type was characterized by strikingly low cognitive control, working memory, episodic memory, and language scores. Moreover, the volumes of the lateral orbitofrontal cortex and inferior temporal gyrus were smaller in the ADHD-C group than in the non-ADHD group (Fig. 2a). Notably, the volume of the right lateral orbitofrontal cortex was positively correlated with language performance in the ADHD-C type (Fig. 2b). These findings specifically indicate anomalies in the right lateral orbitofrontal cortex associated with language deficit in the ADHD-C type. The strength of this study is that, compared to previous studies, we analyzed children with and without ADHD after taking into account various confounding factors. Using data from the ABCD Study, previous studies have successfully identified brain structural differences in children with predisposing factors while controlling for confounding factors such as socioeconomic and pubertal statuses (Bernanke et al., 2022; Hamatani et al., 2022; Hiraoka et al., 2023; Owens et al., 2021). However, there is a paucity of such confounding factors in other studies (e.g. Enhancing Neuroimaging Genetics through Meta-Analysis and ADHD-200). For the first time, we presented cognitive and structural brain characteristics of ADHD subtypes based on cognitive functioning heterogeneity while controlling for various confounding factors.

### Cognitive functioning heterogeneity

We found that the ADHD-A type had higher levels of cognitive control, processing speed, working memory, and episodic memory than the non-ADHD group. Previous studies have reported that patients with ADHD had lower levels of cognitive control (Breitling-Ziegler et al., 2020; Castellanos et al., 2006; Kofler et al., 2019), processing speed (Castellanos et al., 2006; Mohamed et al., 2021), and working memory (Anker et al., 2022; Blomberg et al., 2021) than control participants, which is not supported by our results. Several studies have shown that cognitive function deficits in ADHD may be compensated by better functioning in other cognitive domains, including a high intelligence quotient (Milioni et al., 2017; Ward, Alarcón, Nigg, & Musser, 2015). We suggest that individuals with ADHD-A try to maintain higher cognitive function by employing unique intellectual approaches and cognitive compensatory strategies even in simple tasks.

The ADHD-B type displayed lesser cognitive control, processing speed, and episodic memory than the non-ADHD group, in line with findings of previous studies examining ADHD and cognitive function associations (Breitling-Ziegler et al., 2020; Castellanos et al., 2006; Kofler et al., 2019; Mohamed et al., 2021). Notably, although deficits in working memory have been widely implicated as the core executive function impairment in ADHD (Anker et al., 2022; Blomberg et al., 2021; Castellanos et al., 2006), the present study did not find such impairment in the ADHD-B type. Alternatively, the notable weakness in processing speed may be attributed to the core executive function deficit in the ADHD-B type. Anker et al. (2022) found a correlation between lower levels of processing speed and the attention-deficit in ADHD, suggesting that impaired processing speed is linked to inattentive behavior. As processing speed involves efficient cognitive resource allocation in task performance (Burge et al., 2013; Floyd, Keith, Taub, & McGrew, 2007), individuals with ADHD-B may be particularly vulnerable to cognitive capacity for processing information and generating correct responses within constrained time limits.

Consistent with previous study results (Anker et al., 2022; Blomberg et al., 2019; Blomberg et al., 2021; Breitling-Ziegler et al., 2020; Castellanos et al., 2006; Kofler et al., 2019), the ADHD-C type had poorer cognitive control, working memory, episodic memory, and language abilities than the non-ADHD group did. However, the ADHD-C type did not show any impairment in processing speed. Thus, executive function of the ADHD-C type may be vulnerable to working memory and cognitive control impairments. Working memory is involved in an active mental workspace (Fang et al., 2016) and plays important roles in basic memory maintenance and attention regulation to filter out irrelevant information (Emch, von Bastian, & Koch, 2019). Several studies have shown that working memory capacity is associated with speech production and problem-solving (Emch et al., 2019; Fang et al., 2016). Wiemers and Redick (2018) reported that during the continuous performance test, healthy participants with low working memory capacity had higher selective error and response time to specific stimuli and lower levels of proactive control with more time on the task than those with high working memory capacity, suggesting the involvement of working memory capacity in appropriate cognitive control shifting. We propose that individuals with ADHD-C may be vulnerable to impaired cognitive control and problem-solving abilities owing to their reduced working memory capacity, indicating that individuals with ADHD-C may have difficulties in refusing irrelevant information and cognitive shifting during task performance. These aspects of cognitive functioning heterogeneity may highlight the need for different approaches and considerations for the diagnosis and treatment of ADHD across the identified subtypes.

### Structural characteristics

Structural analyses revealed significantly smaller volumes in the left inferior temporal gyrus and right lateral orbitofrontal cortex in the ADHD-C type than those in the non-ADHD group. Moreover, the ADHD-C type showed a positive correlation between the right lateral orbitofrontal cortex volume and language performance, whereas the volumes in both regions were in the non-ADHD group positively associated with cognitive control, working memory, episodic memory, and language performances. These findings suggest that specific structural anomalies in the right lateral orbitofrontal cortex may underlie language deficits in the ADHD-C type. In contrast, the non-ADHD group exhibited a more widespread association of the left inferior temporal gyrus, in addition to the right lateral orbitofrontal cortex, with various cognitive functions. However, both ADHD-A and ADHD-B types did not show such structural differences in comparison with the non-ADHD group. Therefore, the discussion on structural characteristics is restricted to the ADHD-C type.

Volume reductions in the ADHD-C type were observed in the right lateral orbital part of the inferior frontal gyrus, which is supported by the findings of previous studies on ADHD (Long et al., 2022; Nickel et al., 2018). The lateral orbitofrontal cortex corresponds to cognitive control processes and emotion processing (Deng et al., 2017; Rolls, 2019). Several studies have proposed some functional connections in the lateral orbitofrontal cortex, such that cognitive control is represented predominantly in the frontoparietal network (Lückmann, Jacobs, & Sack, 2014; Tozzi, Goldstein-Piekarski, Korgaonkar, & Williams, 2020), the detection of salient stimuli is associated with the ventral attention network (Corbetta, Patel, & Shulman, 2008; Seeley et al., 2007), and emotional processing is implicated in the subcortical limbic circuit (Banks, Eddy, Angstadt, Nathan, & Phan, 2007), suggesting that this cortex and its association with these networks underlies the functional architecture of cognitive control and emotional processing in ADHD. However, in our study, a significant association between the right lateral orbitofrontal cortex volume and cognitive control was not observed in the ADHD-C type. Instead, this type displayed an association between regional volumes and language abilities. A meta-analytic connectivity modelling study found coactivation of the lateral orbitofrontal cortex with language and auditory processing areas, such as the superior and middle temporal gyri, during tasks involving semantic processing (Zald et al., 2014), indicating the involvement of the lateral orbitofrontal cortex in the language network. This region is known to be involved in anatomical links with Broca's area via ventromedial connections (Miller, Collins, & Kent, 2008). In our study, a volume reduction in the right lateral orbitofrontal cortex and its association with low language performance were specifically seen in the ADHD-C type. This volume reduction observed in individuals with ADHD-C may be associated with language impairment through the abovementioned anomalies in structural and functional connectivity.

Our work provides novel evidence that the ADHD-C type shows decreased left inferior temporal gyrus volume. The inferior temporal gyrus is involved in language processing (Hickok & Poeppel, 2004; Mani et al., 2008) and working memory (Ranganath, Cohen, Dam, & D'Esposito, 2004). Notably, our uncorrected results (*p* < 0.05) indicated a positive correlation between the left inferior temporal gyrus volume and language performance in the ADHD-C type. Although these uncorrected results should be interpreted with caution, the ADHD-C type may induce a volume reduction in the inferior temporal gyrus, leading to impairments in language maintenance, in addition to the volume reduction in the lateral orbitofrontal cortex. In contrast to these structural results, Humphreys et al. (2019) reported that a large GMV of the inferior temporal gyrus is associated with severe ADHD symptoms. This discrepancy might be caused by differences in covariates such as handedness, medication use, household income, and parental education; these factors were not controlled for in their study, which may have weakened the influence of brain structure. Another possible reason is the age difference of the participants. The mean age of patients with the ADHD-C type in our study was 9.8 years. However, in the study by Humphreys et al. (2019), the mean age of the ADHD group was 11.4 years, indicating that the ADHD group may have included more participants who were older than 12 years; notably, developmental delays in several brain regions tend to change at this age (Shaw et al., 2012).

In the non-ADHD group, we found positive correlations between the volumes of the left inferior temporal gyrus and right lateral orbitofrontal cortex and the levels of cognitive control, working memory, episodic memory, and language, indicating that in the non-ADHD group, both brain regions were more closely linked to these cognitive functions. This suggests that the roles of these two brain regions in cognitive function differ between the ADHD-C and non-ADHD groups, although these correlational results might be significant due to the larger sample size of the non-ADHD group compared to that of the ADHD-C group.

Our results, based on FDR-corrected thresholds (*p* < 0.05), also showed that the ADHD-C type had smaller volumes in the left entorhinal cortex, left lateral orbitofrontal cortex, right middle temporal gyrus, right precentral gyrus, right superior frontal gyrus, and right inferior temporal gyrus (Fig. 2 and Table 3). These regions, together with the inferior temporal gyrus and lateral orbitofrontal cortex, are involved in language processing (Behroozmand et al., 2015; He et al., 2013; Hickok & Poeppel, 2004; Lee et al., 2014; Shafritz, Marchione, Gore, Shaywitz, & Shaywitz, 2004), cognitive control (Duan et al., 2018; Lei et al., 2015; Shafritz et al., 2004), and working memory (Buzsáki & Moser, 2013; Duan et al., 2018; Kopniczky et al., 2006). Moreover, some studies have reported associations between these frontal-temporal regional volumes and cognitive functions such as language, cognitive control, and working memory (Behroozmand et al., 2015; Duan et al., 2018; John, Ritter, Wong, & Parks, 2022), suggesting that these structural changes may play an important role in the maintenance of verbal ability, cognitive control, and memory function. Although our FDR-corrected results should be interpreted with caution, they suggest that the ADHD-C type may lead to volume reductions in the entorhinal cortex, middle temporal gyrus, precentral gyrus, and superior frontal gyrus and related cognitive deficits, in addition to volume reductions of the lateral orbitofrontal cortex and inferior temporal gyrus.

Although structural and functional anomalies of the basal ganglia (e.g. reward-related striatum) have often been reported in patients with ADHD (Bonath et al., 2018; Klein et al., 2021; Li et al., 2014; Luo et al., 2023; Moreno-Alcázar et al., 2016; Villemonteix et al., 2015), this study did not find group differences in these regional brain volumes between the ADHD subtypes and the non-ADHD group. One possible reason is the neurobiological difference in ADHD, as previous studies have considered a single ADHD pathology, whereas we distinguished ADHD subtypes based on cognitive function domains, including executive function. Another possible reason is the characteristics of the cognitive function used for clustering: the reward-related task was not included in our clustering method, which may not have identified the reward-related ADHD subtype in the present study. Another possibility is the difference in the sample size and data analysis plan. Previous studies controlled for basic confounders such as age and sex; however, we controlled for additional confounders (e.g. pubertal status, medication use, race/ethnicity, and family conditions).

Considering that a symptom-based diagnosis does not always lead to effective therapy, the National Institute of Mental Health in the United States launched the Research Domain Criteria projects to establish a novel framework for pathophysiological research (Insel & Cuthbert, 2015). Based on this model, the present study aimed to clarify ADHD heterogeneity based on cognitive function domains categorized by unsupervised machine learning and compared brain structural characteristics. The main findings of our study may help establish criteria for more accurate diagnoses based on the underlying ADHD pathophysiology.

Our study has some limitations. First, this study measured the brain volumes of 34 cortical regions labeled with the Desikan atlas and 12 subcortical regions; thus, regional volume measurements may be affected by combined influences of cortical morphology (e.g. close associations with the cortical surface areas). A whole-brain vertex-based analysis might better address the issue of atlas bias, as previously reported (You et al., 2024). Furthermore, cortical thickness, which is used to evaluate cortical maturation abnormalities, may be relatively stable and distinct from heritable brain volume effects (You et al., 2024). Therefore, further analyses as mentioned above are needed. Second, this study was cross-sectional; thus, future longitudinal studies are needed, as previous longitudinal studies have reported differences in atypical brain structures depending on the developmental stage (Hoogman et al., 2019). Third, this study used a restricted cognitive assessment battery measured using the NIH Toolbox. Previous meta-analyses have explored the associations between a broad spectrum of neuropsychological measures (i.e. domains of decision-making, verbal fluency, planning, and vigilance) and ADHD pathology (Frazier, Demaree, & Youngstrom, 2004; Pievsky & McGrath, 2018). It remains unclear how other cognitive domains could have influenced the results of our study. Finally, the ABCD Study data are still being updated and revised with each release, and the data, including sample size, differential diagnosis of psychiatric disorders, clinical measures, neuroimaging, and neuropsychological measures, might differ between previous release versions and the new release version. However, studies using previously released versions of the ABCD Study have built an accumulating body of recent evidence regarding neurobiological mechanisms in ADHD (Kim et al., 2023; Norman et al., 2023; Wang, Zhou, Gui, Liu, & Lu, 2023). Thus, the findings obtained from the ABCD Study should be interpreted with caution, and they should be re-evaluated using revised data in the future.

## Conclusions

To the best of our knowledge, this study is the first to find brain structural anomalies in ADHD subtypes based on cognitive functioning heterogeneity. Behaviorally, ADHD-A was characterized by high levels of cognitive function, ADHD-B by low levels of cognitive control, processing speed, and episodic memory, and ADHD-C showed low levels of cognitive control, working memory, episodic memory, and language abilities. Structurally, only the ADHD-C type exhibited volume reductions in the inferior temporal gyrus and lateral orbitofrontal cortex. Moreover, the volume of the right lateral orbitofrontal cortex was associated with language performance in the ADHD-C type. The present results demonstrate anomalies in the lateral orbitofrontal cortex associated with language deficits in individuals with ADHD-C. Our approach, based on cognitive functioning heterogeneity, may help explain the inconsistencies of previous study findings.

## Supporting information

Yamashita et al. supplementary material Yamashita et al. supplementary material

## Data Availability

Data can be accessed through registration with the ABCD Study at https://nda.nih.gov/abcd. The data used in this manuscript are available from the ABCD Study's Data Release 2.0 (https://doi.org/10.15154/1504041). Information on how to access ABCD data through the NDA is available on the ABCD Study data-sharing webpage: https://abcdstudy.org/scientists_data_sharing.html. Instructions on how to create an NDA study are available at https://nda.nih.gov/nda/webinars-and-tutorials. R codes for this analysis are available at https://osf.io/nvwam/ after acceptance.

## Supplementary material

The supplementary material for this article can be found at https://doi.org/10.1017/S0033291724002368

## References

[ref3] Achenbach, T. M., & Rescorla, L. A. (2001). Manual for the ASEBA school-age forms & profiles. Burlington, VT: University of Vermont, Research Center for Children, Youth, & Families.

[ref4] American Psychiatric Association (2013). Diagnostic and statistical manual of mental disorders (5th ed.). Washington, DC: American Psychiatric Publishing.

[ref5] Anker, E., Ogrim, G., & Heir, T. (2022). Verbal working memory and processing speed: Correlations with the severity of attention deficit and emotional dysregulation in adult ADHD. Journal of Neuropsychology, 16(1), 211–235. doi: 10.1111/jnp.12260 34218514 PMC9290636

[ref6] Ball, G. H., & Hall, D. J. (1965). ISODATA, a novel method of data analysis and pattern classification. Menlo Park, CA: Stanford Research Institute.

[ref7] Banks, S. J., Eddy, K. T., Angstadt, M., Nathan, P. J., & Phan, K. L. (2007). Amygdala-frontal connectivity during emotion regulation. Social Cognitive and Affective Neuroscience, 2(4), 303–312. doi: 10.1093/scan/nsm029 18985136 PMC2566753

[ref8] Barkley, R. A. (1997). Behavioral inhibition, sustained attention, and executive functions: Constructing a unifying theory of ADHD. Psychological Bulletin, 121(1), 65–94. doi: 10.1037/0033-2909.121.1.65 9000892

[ref9] Behroozmand, R., Shebek, R., Hansen, D. R., Oya, H., Robin, D. A., Howard, III. M. A., & Greenlee, J. D. (2015). Sensory-motor networks involved in speech production and motor control: An fMRI study. NeuroImage, 109, 418–428. doi: 10.1016/j.neuroimage.2015.01.040 25623499 PMC4339397

[ref10] Bernanke, J., Luna, A., Chang, L., Bruno, E., Dworkin, J., & Posner, J. (2022). Structural brain measures among children with and without ADHD in the adolescent brain and cognitive development study cohort: A cross-sectional US population-based study. The Lancet. Psychiatry, 9(3), 222–231. doi: 10.1016/S2215-0366(21)00505-8 35143759

[ref11] Blomberg, R., Danielsson, H., Rudner, M., Söderlund, G. B. W., & Rönnberg, J. (2019). Speech processing difficulties in attention deficit hyperactivity disorder. Frontiers in Psychology, 10, 1536. doi: 10.3389/fpsyg.2019.01536 31333549 PMC6624822

[ref12] Blomberg, R., Johansson Capusan, A., Signoret, C., Danielsson, H., & Rönnberg, J. (2021). The effects of working memory load on auditory distraction in adults with attention deficit hyperactivity disorder. Frontiers in Human Neuroscience, 15, 771711. doi: 10.3389/fnhum.2021.771711 34916918 PMC8670091

[ref13] Bonath, B., Tegelbeckers, J., Wilke, M., Flechtner, H. H., & Krauel, K. (2018). Regional gray matter volume differences between adolescents with ADHD and typically developing controls: Further evidence for anterior cingulate involvement. Journal of Attention Disorders, 22(7), 627–638. doi: 10.1177/1087054715619682 26748338

[ref14] Breitling-Ziegler, C., Tegelbeckers, J., Flechtner, H. H., & Krauel, K. (2020). Economical assessment of working memory and response inhibition in ADHD using a combined *n*-back/Nogo paradigm: An ERP study. Frontiers in Human Neuroscience, 14, 322. doi: 10.3389/fnhum.2020.00322 32848679 PMC7426064

[ref15] Burge, W. K., Ross, L. A., Amthor, F. R., Mitchell, W. G., Zotov, A., & Visscher, K. M. (2013). Processing speed training increases the efficiency of attentional resource allocation in young adults. Frontiers in Human Neuroscience, 7, 684. doi: 10.3389/fnhum.2013.00684 24151461 PMC3799007

[ref16] Buzsáki, G., & Moser, E. I. (2013). Memory, navigation and theta rhythm in the hippocampal-entorhinal system. Nature Neuroscience, 16(2), 130–138. doi: 10.1038/nn.3304 23354386 PMC4079500

[ref17] Cai, W., Warren, S. L., Duberg, K., Yu, A., Hinshaw, S. P., & Menon, V. (2023). Both reactive and proactive control are deficient in children with ADHD and predictive of clinical symptoms. Translational Psychiatry, 13(1), 179. doi: 10.1038/s41398-023-02471-w 37236924 PMC10220086

[ref2] Casey, B. J., Cannonier, T., Conley, M. I., Cohen, A. O., Barch, D. M., Heitzeg, M. M., … ABCD Imaging Acquisition Workgroup. (2018). The Adolescent Brain Cognitive Development (ABCD) study: Imaging acquisition across 21 sites. Developmental Cognitive Neuroscience, 32, 43–54. doi: 10.1016/j.dcn.2018.03.001 29567376 PMC5999559

[ref18] Castellanos, F. X., Sonuga-Barke, E. J., Milham, M. P., & Tannock, R. (2006). Characterizing cognition in ADHD: Beyond executive dysfunction. Trends in Cognitive Sciences, 10(3), 117–123. doi: 10.1016/j.tics.2006.01.011 16460990

[ref19] Cheng, W., Rolls, E., Gong, W., Du, J., Zhang, J., Zhang, X. Y., … Feng, J. (2021). Sleep duration, brain structure, and psychiatric and cognitive problems in children. Molecular Psychiatry, 26(8), 3992–4003. doi: 10.1038/s41380-020-0663-2 32015467 PMC8855973

[ref20] Corbetta, M., Patel, G., & Shulman, G. L. (2008). The reorienting system of the human brain: From environment to theory of mind. Neuron, 58(3), 306–324. doi: 10.1016/j.neuron.2008.04.017 18466742 PMC2441869

[ref21] Deng, W., Rolls, E. T., Ji, X., Robbins, T. W., Banaschewski, T., Bokde, A. L. W., … Feng, J. (2017). Separate neural systems for behavioral change and for emotional responses to failure during behavioral inhibition. Human Brain Mapping, 38(7), 3527–3537. doi: 10.1002/hbm.23607 28429498 PMC6866896

[ref22] Duan, K., Chen, J., Calhoun, V. D., Lin, D., Jiang, W., Franke, B., … Liu, J. (2018). Neural correlates of cognitive function and symptoms in attention-deficit/hyperactivity disorder in adults. NeuroImage. Clinical, 19, 374–383. doi: 10.1016/j.nicl.2018.04.035 30013920 PMC6044210

[ref23] Emch, M., von Bastian, C. C., & Koch, K. (2019). Neural correlates of verbal working memory: An fMRI meta-analysis. Frontiers in Human Neuroscience, 13, 180. doi: 10.3389/fnhum.2019.00180 31244625 PMC6581736

[ref24] Fang, X., Zhang, Y., Zhou, Y., Cheng, L., Li, J., Wang, Y., … Jiang, T. (2016). Resting-state coupling between core regions within the central-executive and salience networks contributes to working memory performance. Frontiers in Behavioral Neuroscience, 10, 27. doi: 10.3389/fnbeh.2016.00027 26941629 PMC4766291

[ref25] Feldman, H. M., & Reiff, M. I. (2014). Clinical practice. Attention deficit-hyperactivity disorder in children and adolescents. The New England Journal of Medicine, 370(9), 838–846. doi: 10.1056/NEJMcp1307215 24571756

[ref26] Floyd, R. G., Keith, T. Z., Taub, G. E., & McGrew, K. S. (2007). Cattell-Horn-Carroll cognitive abilities and their effects on reading decoding skills: *G* has indirect effects, more specific abilities have direct effects. School Psychology Quarterly, 22(2), 200–233. doi: 10.1037/1045-3830.22.2.200

[ref27] Fox, R. S., Manly, J. J., Slotkin, J., Devin Peipert, J., & Gershon, R. C. (2021). Reliability and validity of the Spanish-language version of the NIH toolbox. Assessment, 28(2), 457–471. doi: 10.1177/1073191120913943 32264689 PMC7541574

[ref28] Frazier, T. W., Demaree, H. A., & Youngstrom, E. A. (2004). Meta-analysis of intellectual and neuropsychological test performance in attention-deficit/hyperactivity disorder. Neuropsychology, 18(3), 543–555. doi: 10.1037/0894-4105.18.3.543 15291732

[ref29] Friedman, N. P., Miyake, A., Young, S. E., DeFries, J. C., Corley, R. P., & Hewitt, J. K. (2008). Individual differences in executive functions are almost entirely genetic in origin. Journal of Experimental Psychology. General, 137(2), 201–225. doi: 10.1037/0096-3445.137.2.201 18473654 PMC2762790

[ref30] Garavan, H., Bartsch, H., Conway, K., Decastro, A., Goldstein, R. Z., Heeringa, S., … Zahs, D. (2018). Recruiting the ABCD sample: Design considerations and procedures. Developmental Cognitive Neuroscience, 32, 16–22. doi: 10.1016/j.dcn.2018.04.004 29703560 PMC6314286

[ref31] Hagler, Jr. D. J., Hatton, S., Cornejo, M. D., Makowski, C., Fair, D. A., Dick, A. S., … Dale, A. M. (2019). Image processing and analysis methods for the Adolescent Brain Cognitive Development study. NeuroImage, 202, 116091. doi: 10.1016/j.neuroimage.2019.116091 31415884 PMC6981278

[ref32] Hamatani, S., Hiraoka, D., Makita, K., Tomoda, A., & Mizuno, Y. (2022). Longitudinal impact of COVID-19 pandemic on mental health of children in the ABCD study cohort. Scientific Reports, 12(1), 19601. doi: 10.1038/s41598-022-22694-z 36379997 PMC9665012

[ref33] He, Q., Xue, G., Chen, C., Chen, C., Lu, Z. L., & Dong, Q. (2013). Decoding the neuroanatomical basis of reading ability: A multivoxel morphometric study. The Journal of Neuroscience: the Official Journal of the Society for Neuroscience, 33(31), 12835–12843. doi: 10.1523/JNEUROSCI.0449-13.2013 23904618 PMC3728691

[ref34] Heeringa, S., & Berglund, P. A. (2020). A guide for population-based analysis of the Adolescent Brain Cognitive Development (ABCD) study baseline data. Preprint at 10.1101/2020.02.10.942011

[ref35] Hickok, G., & Poeppel, D. (2004). Dorsal and ventral streams: A framework for understanding aspects of the functional anatomy of language. Cognition, 92(1–2), 67–99. doi: 10.1016/j.cognition.2003.10.011 15037127

[ref36] Hiraoka, D., Makita, K., Hamatani, S., Tomoda, A., & Mizuno, Y. (2023). Effects of prenatal cannabis exposure on developmental trajectory of cognitive ability and brain volumes in the Adolescent Brain Cognitive Development (ABCD) study. Developmental Cognitive Neuroscience, 60, 101209. doi: 10.1016/j.dcn.2023.101209 36791556 PMC9950823

[ref37] Hoogman, M., Muetzel, R., Guimaraes, J. P., Shumskaya, E., Mennes, M., Zwiers, M. P., … Franke, B. (2019). Brain imaging of the cortex in ADHD: A coordinated analysis of large-scale clinical and population-based samples. The American Journal of Psychiatry, 176(7), 531–542. doi: 10.1176/appi.ajp.2019.18091033 31014101 PMC6879185

[ref38] Humphreys, K. L., Watts, E. L., Dennis, E. L., King, L. S., Thompson, P. M., & Gotlib, I. H. (2019). Stressful life events, ADHD symptoms, and brain structure in early adolescence. Journal of Abnormal Child Psychology, 47(3), 421–432. doi: 10.1007/s10802-018-0443-5 29785533 PMC6249129

[ref39] Insel, T. R., & Cuthbert, B. N. (2015). Medicine. Brain disorders? Precisely. Science *(*New York, N.Y.*)*, 348(6234), 499–500. doi: 10.1126/science.aab2358 25931539

[ref1] Jernigan, T. L., Brown, S. A., & ABCD Consortium Coordinators. (2018). Introduction. Developmental Cognitive Neuroscience, 32, 1–3. doi: 10.1016/j.dcn.2018.02.002 29496476 PMC6969247

[ref40] John, S. E., Ritter, A., Wong, C., & Parks, C. M. (2022). The roles of executive functioning, simple attention, and medial temporal lobes in early learning, late learning, and delayed recall. Neuropsychology, Development, and Cognition. Section B, Aging, Neuropsychology and Cognition, 29(3), 400–417. doi: 10.1080/13825585.2021.2016583 PMC8960335 34919026

[ref41] Kim, W. P., Kim, H. J., Pack, S. P., Lim, J. H., Cho, C. H., & Lee, H. J. (2023). Machine learning-based prediction of attention-deficit/hyperactivity disorder and sleep problems with wearable data in children. JAMA Network Open, 6(3), e233502. doi: 10.1001/jamanetworkopen.2023.3502 36930149 PMC10024208

[ref42] Klein, M., Souza-Duran, F. L., Menezes, A. K. P. M., Alves, T. M., Busatto, G., & Louzã, M. R. (2021). Gray matter volume in elderly adults with ADHD: Associations of symptoms and comorbidities with brain structures. Journal of Attention Disorders, 25(6), 829–838. doi: 10.1177/1087054719855683 31262214

[ref43] Kobak, K. A., Kratochvil, C. J., Stanger, C., & Kaufman, J. (2013). Computerized screening of comorbidity in adolescents with substance or psychiatric disorders. Paper presented at 2013 *Anxiety and Depression: Technology and New Media in Practice and Research* (*La Jolaa, CA*).

[ref44] Kofler, M. J., Irwin, L. N., Soto, E. F., Groves, N. B., Harmon, S. L., & Sarver, D. E. (2019). Executive functioning heterogeneity in pediatric ADHD. Journal of Abnormal Child Psychology, 47(2), 273–286. doi: 10.1007/s10802-018-0438-2 29705926 PMC6204311

[ref45] Kopniczky, Z., Dochnal, R., Mácsai, M., Pál, A., Kiss, G., Mihály, A., & Szabo, G. (2006). Alterations of behavior and spatial learning after unilateral entorhinal ablation of rats. Life Sciences, 78(23), 2683–2688. doi: 10.1016/j.lfs.2005.10.014 16313927

[ref46] Lacerda, B. C., Martínez, S. B. S., Franz, A. P., Moreira-Maia, C. R., Silveira, R. C., Procianoy, R. S., … Wagner, F. (2020). Does ADHD worsen inhibitory control in preschool children born very premature and/or with very low birth weight? Trends in Psychiatry and Psychotherapy, 42(4), 340–347. doi: 10.1590/2237-6089-2019-0075 33263709 PMC7879083

[ref47] Lee, N. R., Raznahan, A., Wallace, G. L., Alexander-Bloch, A., Clasen, L. S., Lerch, J. P., & Giedd, J. N. (2014). Anatomical coupling among distributed cortical regions in youth varies as a function of individual differences in vocabulary abilities. Human Brain Mapping, 35(5), 1885–1895. doi: 10.1002/hbm.22299 23728856 PMC6869329

[ref48] Lei, D., Du, M., Wu, M., Chen, T., Huang, X., Du, X., … Gong, Q. (2015). Functional MRI reveals different response inhibition between adults and children with ADHD. Neuropsychology, 29(6), 874–881. doi: 10.1037/neu0000200 25938917

[ref49] Li, F., He, N., Li, Y., Chen, L., Huang, X., Lui, S., … Gong, Q. (2014). Intrinsic brain abnormalities in attention deficit hyperactivity disorder: A resting-state functional MR imaging study. Radiology, 272(2), 514–523. doi: 10.1148/radiol.14131622 24785156

[ref50] Long, Y., Pan, N., Ji, S., Qin, K., Chen, Y., Zhang, X., … Gong, Q. (2022). Distinct brain structural abnormalities in attention-deficit/hyperactivity disorder and substance use disorders: A comparative meta-analysis. Translational Psychiatry, 12(1), 368. doi: 10.1038/s41398-022-02130-6 36068207 PMC9448791

[ref51] Lückmann, H. C., Jacobs, H. I., & Sack, A. T. (2014). The cross-functional role of frontoparietal regions in cognition: Internal attention as the overarching mechanism. Progress in Neurobiology, 116, 66–86. doi: 10.1016/j.pneurobio.2014.02.002 24530293

[ref52] Luo, L., You, W., DelBello, M. P., Gong, Q., & Li, F. (2022). Recent advances in psychoradiology. Physics in Medicine and Biology, 67(23), 23TR01. doi: 10.1088/1361-6560/ac9d1e 36279868

[ref53] Luo, L., Chen, L., Wang, Y., Li, Q., He, N., Li, Y., … Li, F. (2023). Patterns of brain dynamic functional connectivity are linked with attention-deficit/hyperactivity disorder-related behavioral and cognitive dimensions. Psychological Medicine, 53(14), 6666–6677. doi: 10.1017/S0033291723000089 PMC10600939 36748350

[ref54] Ma, Q., Wang, H., Rolls, E. T., Xiang, S., Li, J., Li, Y., … Li, F. (2022). Lower gestational age is associated with lower cortical volume and cognitive and educational performance in adolescence. BMC Medicine, 20(1), 424. doi: 10.1186/s12916-022-02627-3 36329481 PMC9635194

[ref55] Mani, J., Diehl, B., Piao, Z., Schuele, S. S., Lapresto, E., Liu, P., … Lüders, H. O. (2008). Evidence for a basal temporal visual language center: Cortical stimulation producing pure alexia. Neurology, 71(20), 1621–1627. doi: 10.1212/01.wnl.0000334755.32850.f0 19001252

[ref56] Milioni, A. L., Chaim, T. M., Cavallet, M., de Oliveira, N. M., Annes, M., Dos Santos, B., … Cunha, P. J. (2017). High IQ may “mask” the diagnosis of ADHD by compensating for deficits in executive functions in treatment-naïve adults with ADHD. Journal of Attention Disorders, 21(6), 455–464. doi: 10.1177/1087054714554933 25359760

[ref57] Miller, L. A., Collins, R. L., & Kent, T. A. (2008). Language and the modulation of impulsive aggression. The Journal of Neuropsychiatry and Clinical Neurosciences, 20(3), 261–273. doi: 10.1176/jnp.2008.20.3.261 18806230

[ref58] Miyake, A., Friedman, N. P., Emerson, M. J., Witzki, A. H., Howerter, A., & Wager, T. D. (2000). The unity and diversity of executive functions and their contributions to complex “frontal lobe” tasks: A latent variable analysis. Cognitive Psychology, 41(1), 49–100. doi: 10.1006/cogp.1999.0734 10945922

[ref59] Mohamed, S. M. H., Butzbach, M., Fuermaier, A. B. M., Weisbrod, M., Aschenbrenner, S., Tucha, L., & Tucha, O. (2021). Basic and complex cognitive functions in adult ADHD. PLoS One, 16(9), e0256228. doi: 10.1371/journal.pone.0256228 34473722 PMC8412315

[ref60] Moreno-Alcázar, A., Ramos-Quiroga, J. A., Radua, J., Salavert, J., Palomar, G., Bosch, R., … Clotet, E. (2016). Brain abnormalities in adults with attention deficit hyperactivity disorder revealed by voxel-based morphometry. Psychiatry Research. Neuroimaging, 254, 41–47. doi: 10.1016/j.pscychresns.2016.06.002 27318593

[ref61] Nakao, T., Radua, J., Rubia, K., & Mataix-Cols, D. (2011). Gray matter volume abnormalities in ADHD: Voxel-based meta-analysis exploring the effects of age and stimulant medication. The American Journal of Psychiatry, 168(11), 1154–1163. doi: 10.1176/appi.ajp.2011.11020281 21865529

[ref62] Nickel, K., Tebartz van Elst, L., Manko, J., Unterrainer, J., Rauh, R., Klein, C., … Maier, S. (2018). Inferior frontal gyrus volume loss distinguishes between autism and (comorbid) attention-deficit/hyperactivity disorder—A FreeSurfer analysis in children. Frontiers in Psychiatry, 9, 521. doi: 10.3389/fpsyt.2018.00521 30405459 PMC6206215

[ref63] Nolin, S. A., Cowart, H., Merritt, S., McInerney, K., Bharadwaj, P. K., Franchetti, M. K., … Visscher, K. M. (2023). Validity of the NIH toolbox cognitive battery in a healthy oldest-old 85+ sample. Journal of the International Neuropsychological Society: JINS, 29(6), 605–614. doi: 10.1017/S1355617722000443 36239453 PMC11172394

[ref64] Norman, L. J., Price, J., Ahn, K., Sudre, G., Sharp, W., & Shaw, P. (2023). Longitudinal trajectories of childhood and adolescent attention deficit hyperactivity disorder diagnoses in three cohorts. EClinicalMedicine, 60, 102021. doi: 10.1016/j.eclinm.2023.102021 37333663 PMC10272308

[ref65] Ott, L. R., Schantell, M., Willett, M. P., Johnson, H. J., Eastman, J. A., Okelberry, H. J., … May, P. E. (2022). Construct validity of the NIH toolbox cognitive domains: A comparison with conventional neuropsychological assessments. Neuropsychology, 36(5), 468–481. doi: 10.1037/neu0000813 35482626 PMC10468104

[ref66] Owens, M. M., Allgaier, N., Hahn, S., Yuan, D., Albaugh, M., Adise, S., … Garavan, H. (2021). Multimethod investigation of the neurobiological basis of ADHD symptomatology in children aged 9-10: Baseline data from the ABCD study. Translational Psychiatry, 11(1), 64. doi: 10.1038/s41398-020-01192-8 33462190 PMC7813832

[ref67] Paul, S. E., Hatoum, A. S., Fine, J. D., Johnson, E. C., Hansen, I., Karcher, N. R., … Bogdan, R. (2021). Associations between prenatal cannabis exposure and childhood outcomes: Results from the ABCD study. JAMA Psychiatry, 78(1), 64–76. doi: 10.1001/jamapsychiatry.2020.2902 32965490 PMC7512132

[ref68] Petersen, A. C., Crockett, L., Richards, M., & Boxer, A. (1988). A self-report measure of pubertal status: Reliability, validity, and initial norms. Journal of Youth and Adolescence, 17(2), 117–133. doi: 10.1007/BF01537962 24277579

[ref69] Pievsky, M. A., & McGrath, R. E. (2018). The neurocognitive profile of attention-deficit/hyperactivity disorder: A review of meta-analyses. Archives of Clinical Neuropsychology: The Official Journal of the National Academy of Neuropsychologists, 33(2), 143–157. doi: 10.1093/arclin/acx055 29106438

[ref70] Postema, M. C., Hoogman, M., Ambrosino, S., Asherson, P., Banaschewski, T., Bandeira, C. E., … Francks, C. (2021). Analysis of structural brain asymmetries in attention-deficit/hyperactivity disorder in 39 datasets. Journal of Child Psychology and Psychiatry, and Allied Disciplines, 62(10), 1202–1219. doi: 10.1111/jcpp.13396 33748971 PMC8455726

[ref71] Qureshi, M. N., Min, B., Jo, H. J., & Lee, B. (2016). Multiclass classification for the differential diagnosis on the ADHD subtypes using recursive feature elimination and hierarchical extreme learning machine: Structural MRI study. PLoS One, 11(8), e0160697. doi: 10.1371/journal.pone.0160697 27500640 PMC4976974

[ref72] Ranganath, C., Cohen, M. X., Dam, C., & D'Esposito, M. (2004). Inferior temporal, prefrontal, and hippocampal contributions to visual working memory maintenance and associative memory retrieval. The Journal of Neuroscience: The Official Journal of the Society for Neuroscience, 24(16), 3917–3925. doi: 10.1523/JNEUROSCI.5053-03.2004 15102907 PMC6729418

[ref73] Rolls, E. T. (2019). The orbitofrontal cortex and emotion in health and disease, including depression. Neuropsychologia, 128, 14–43. doi: 10.1016/j.neuropsychologia.2017.09.021 28951164

[ref74] Saad, J. F., Griffiths, K. R., & Korgaonkar, M. S. (2020). A systematic review of imaging studies in the combined and inattentive subtypes of attention deficit hyperactivity disorder. Frontiers in Integrative Neuroscience, 14, 31. doi: 10.3389/fnint.2020.00031 32670028 PMC7327109

[ref75] Salari, R., Bohlin, G., Rydell, A. M., & Thorell, L. B. (2017). Neuropsychological functioning and attachment representations in early school age as predictors of ADHD symptoms in late adolescence. Child Psychiatry and Human Development, 48(3), 370–384. doi: 10.1007/s10578-016-0664-1 27349655

[ref76] Scheerer, N. E., Curcin, K., Stojanoski, B., Anagnostou, E., Nicolson, R., Kelley, E., … Stevenson, R. A. (2021). Exploring sensory phenotypes in autism spectrum disorder. Molecular Autism, 12(1), 67. doi: 10.1186/s13229-021-00471-5 34641960 PMC8507349

[ref77] Seeley, W. W., Menon, V., Schatzberg, A. F., Keller, J., Glover, G. H., Kenna, H., … Greicius, M. D. (2007). Dissociable intrinsic connectivity networks for salience processing and executive control. The Journal of Neuroscience: The Official Journal of the Society for Neuroscience, 27(9), 2349–2356. doi: 10.1523/JNEUROSCI.5587-06.2007 17329432 PMC2680293

[ref78] Seidman, L. J., Biederman, J., Liang, L., Valera, E. M., Monuteaux, M. C., Brown, A., … Makris, N. (2011). Gray matter alterations in adults with attention-deficit/hyperactivity disorder identified by voxel based morphometry. Biological Psychiatry, 69(9), 857–866. doi: 10.1016/j.biopsych.2010.09.053 21183160 PMC3940267

[ref79] Semrud-Clikeman, M., Pliszka, S. R., Bledsoe, J., & Lancaster, J. (2014). Volumetric MRI differences in treatment naïve and chronically treated adolescents with ADHD-combined type. Journal of Attention Disorders, 18(6), 511–520. doi: 10.1177/1087054712443158 22653807

[ref80] Serrallach, B. L., Groß, C., Christiner, M., Wildermuth, S., & Schneider, P. (2022). Neuromorphological and neurofunctional correlates of ADHD and ADD in the auditory cortex of adults. Frontiers in Neuroscience, 16, 850529. doi: 10.3389/fnins.2022.850529 35600622 PMC9121124

[ref81] Shafritz, K. M., Marchione, K. E., Gore, J. C., Shaywitz, S. E., & Shaywitz, B. A. (2004). The effects of methylphenidate on neural systems of attention in attention deficit hyperactivity disorder. The American Journal of Psychiatry, 161(11), 1990–1997. doi: 10.1176/appi.ajp.161.11.1990 15514398

[ref82] Shaw, P., Malek, M., Watson, B., Sharp, W., Evans, A., & Greenstein, D. (2012). Development of cortical surface area and gyrification in attention-deficit/hyperactivity disorder. Biological Psychiatry, 72(3), 191–197. doi: 10.1016/j.biopsych.2012.01.031 22418014 PMC10376909

[ref83] Thomas, R., Sanders, S., Doust, J., Beller, E., & Glasziou, P. (2015). Prevalence of attention-deficit/hyperactivity disorder: A systematic review and meta-analysis. Pediatrics, 135(4), e994–e1001. doi: 10.1542/peds.2014-3482 25733754

[ref84] Tozzi, L., Goldstein-Piekarski, A. N., Korgaonkar, M. S., & Williams, L. M. (2020). Connectivity of the cognitive control network during response inhibition as a predictive and response biomarker in major depression: Evidence from a randomized clinical trial. Biological Psychiatry, 87(5), 462–472. doi: 10.1016/j.biopsych.2019.08.005 31601424 PMC8628639

[ref85] van Rossum, M. C., da Silva, P. M. A., Wang, Y., Kouwenhoven, E. A., & Hermens, H. J. (2023). Missing data imputation techniques for wireless continuous vital signs monitoring. Journal of Clinical Monitoring and Computing, 37(5), 1387–1400. doi: 10.1007/s10877-023-00975-w 36729298 PMC9893204

[ref86] Villemonteix, T., De Brito, S. A., Kavec, M., Balériaux, D., Metens, T., Slama, H., … Massat, I. (2015). Grey matter volumes in treatment naïve vs. chronically treated children with attention deficit/hyperactivity disorder: A combined approach. European Neuropsychopharmacology: The Journal of the European College of Neuropsychopharmacology, 25(8), 1118–1127. doi: 10.1016/j.euroneuro.2015.04.015 25934396

[ref87] Wang, Z., Zhou, X., Gui, Y., Liu, M., & Lu, H. (2023). Multiple measurement analysis of resting-state fMRI for ADHD classification in adolescent brain from the ABCD study. Translational Psychiatry, 13(1), 45. doi: 10.1038/s41398-023-02309-5 36746929 PMC9902465

[ref88] Ward, A. R., Alarcón, G., Nigg, J. T., & Musser, E. D. (2015). Variation in parasympathetic dysregulation moderates short-term memory problems in childhood attention-deficit/hyperactivity disorder. Journal of Abnormal Child Psychology, 43(8), 1573–1583. doi: 10.1007/s10802-015-0054-3 26216249 PMC4625793

[ref89] Wiemers, E. A., & Redick, T. S. (2018). Working memory capacity and intra-individual variability of proactive control. Acta Psychologica, 182, 21–31. doi: 10.1016/j.actpsy.2017.11.002 29127776 PMC5771817

[ref90] Wiker, T., Norbom, L. B., Beck, D., Agartz, I., Andreassen, O. A., Alnæs, D., … Tamnes, C. K. (2023). Reaction time variability in children is specifically associated with attention problems and regional white matter microstructure. Biological Psychiatry. Cognitive Neuroscience and Neuroimaging, 8(8), 832–840. doi: 10.1016/j.bpsc.2023.03.010 37003411

[ref91] Willcutt, E. G., Doyle, A. E., Nigg, J. T., Faraone, S. V., & Pennington, B. F. (2005). Validity of the executive function theory of attention-deficit/hyperactivity disorder: A meta-analytic review. Biological Psychiatry, 57(11), 1336–1346. doi: 10.1016/j.biopsych.2005.02.006 15950006

[ref92] Williams, M. C., Bednarski, B. P., Pieszko, K., Miller, R. J. H., Kwiecinski, J., Shanbhag, A., … Slomka, P. J. (2023). Unsupervised learning to characterize patients with known coronary artery disease undergoing myocardial perfusion imaging. European Journal of Nuclear Medicine and Molecular Imaging, 50(9), 2656–2668. doi: 10.1007/s00259-023-06218-z 37067586 PMC10317876

[ref93] Wu, Z. M., Llera, A., Hoogman, M., Cao, Q. J., Zwiers, M. P., Bralten, J., … Wang, Y. F. (2019). Linked anatomical and functional brain alterations in children with attention-deficit/hyperactivity disorder. Neuroimage. Clinical, 23, 101851. doi: 10.1016/j.nicl.2019.101851 31077980 PMC6514365

[ref94] You, W., Li, Q., Chen, L., He, N., Li, Y., Long, F., … Li, F. (2024). Common and distinct cortical thickness alterations in youth with autism spectrum disorder and attention-deficit/hyperactivity disorder. BMC Medicine, 22(1), 92. doi: 10.1186/s12916-024-03313-2 38433204 PMC10910790

[ref95] Yu, W., Wang, Q., Ge, M., & Shi, X. (2022). Cluster analysis of lymphocyte subset from peripheral blood in newly diagnosed idiopathic aplastic anaemia patients. Annals of Medicine, 54(1), 2431–2439. doi: 10.1080/07853890.2022.2118367 36066098 PMC9481148

[ref96] Zald, D. H., McHugo, M., Ray, K. L., Glahn, D. C., Eickhoff, S. B., & Laird, A. R. (2014). Meta-analytic connectivity modeling reveals differential functional connectivity of the medial and lateral orbitofrontal cortex. Cerebral Cortex *(*New York, N.Y.: 1991*)*, 24(1), 232–248. doi: 10.1093/cercor/bhs308 23042731 PMC3862271

[ref97] Zhang, M., Huang, Y., Jiao, J., Yuan, D., Hu, X., Yang, P., … Huang, Y. (2022a). Transdiagnostic symptom subtypes across autism spectrum disorders and attention deficit hyperactivity disorder: Validated by measures of neurocognition and structural connectivity. BMC Psychiatry, 22(1), 102. doi: 10.1186/s12888-022-03734-4 35139813 PMC8827180

[ref98] Zhang, R., Manza, P., & Volkow, N. D. (2022b). Prenatal caffeine exposure: Association with neurodevelopmental outcomes in 9- to 11-year-old children. Journal of Child Psychology and Psychiatry, and Allied Disciplines, 63(5), 563–578. doi: 10.1111/jcpp.13495 34318489 PMC9291501

[ref99] Zhao, Q., Voon, V., Zhang, L., Shen, C., Zhang, J., & Feng, J. (2022). The ABCD study: Brain heterogeneity in intelligence during a neurodevelopmental transition stage. Cerebral Cortex *(*New York, N.Y.: 1991*)*, 32(14), 3098–3109. doi: 10.1093/cercor/bhab403 35037940 PMC9290553

