# Supplementary material for: Unsupervised machine learning for identifying attention-deficit/hyperactivity disorder subtypes based on cognitive function and their implications for brain structure
Source: Psychol Med. 2024 Sep 26;54(14):3917–29. doi: 10.1017/S0033291724002368 (PMC11578918; doi:10.1017/S0033291724002368)
Supplement: Yamashita et al. supplementary material [file S0033291724002368sup001.docx]

**Online Supplementary Material**

**Supplementary Methods 1. National Institutes of Health Toolbox Tasks**

In the flanker inhibitory control and attention task, participants were instructed to indicate the left-right orientation of a central arrow flanked by two arrows pointing in the same (congruent) or different (incongruent) direction. In the dimensional change and sort task, participants were instructed to match a series of bivalent test pictures (e.g., blue truck and yellow ball) first according to one dimension (e.g., colour) and then according to the other (e.g., shape). Additionally, participants were asked to change the dimension being matched and choose the correct image during switch trials. In the pattern comparison processing speed task, participants were instructed to use their dominant hand to tap “yes” if the stimuli presented on the screen were the same and “no” if they were not. In the list sorting working memory task, participants were instructed to repeat the presented list of items in order from smallest to largest according to one dimension (e.g., animals) and then according to both dimensions (e.g., first animals, then food). In the picture sequence memory task, images and verbal statements of events (e.g., “going to the park”) were presented sequentially and assigned the corresponding positions on the screen. Subsequently, images were presented scrambled in the screen centre, and participants were instructed to replicate the sequence by dragging the images to their appropriate positions. In the picture vocabulary task, participants were instructed to select an image from a set of four that corresponded to a read-out word. In the oral reading recognition task, participants were instructed to read out words presented on the screen.

**Supplementary Methods 2. A linear mixed-effects model adjusted for comorbidities**

The attention-deficit/hyperactivity disorder (ADHD) groups in this study included some comorbidities (e.g., symptoms of depression and anxiety), because most individuals with ADHD have at least one psychiatric comorbidity (Bishop, Mulraney, Rinehart, & Sciberras, 2019; Seo et al., 2022; Tistarelli, Fagnani, Troianiello, Stazi, & Adriani, 2020; Wu, Joubran, Kumar, Assadi, & Nguyen, 2023). However, this limits the ability to generalize the reported cognitive and neuroimaging results to ADHD without comorbidities. Thus, we investigated additional linear mixed-effects models adjusted for comorbidities. Specifically, we investigated cognitive function features by comparing each ADHD group with the non-ADHD group as follows. A linear mixed-effects model was used with each cognitive function as the dependent variable and the group as the independent variable. Based on previous studies (Hamatani, Hiraoka, Makita, Tomoda, & Mizuno, 2022; Hiraoka, Makita, Hamatani, Tomoda, & Mizuno, 2023), family ID (sibling status), multiple data correction sites, and twin or triplet status were modelled as random effects. Covariates included comorbidities, such as depressive disorder, anxiety disorder, conduct disorder, and oppositional defiant disorder, based on Kiddie Schedule for Affective Disorders and Schizophrenia (K-SADS) from parents (1, diagnosed; 0, undiagnosed) in addition to the variables described in the main text (see “Demographic variables and covariates” in the “Methods” section). Additionally, to test brain structural characteristics by comparing each ADHD subtype with the non-ADHD group, an additional linear mixed-effects model was used with each regional brain volume as the dependent variable and group as the independent variable. In addition to multiple data collection sites and twin or triplet status, we included family ID as a random effect nested inside a random effect of the magnetic resonance imaging (MRI) scanner to account for the large number of siblings and multiple data collection sites, as previously recommended (Bernanke et al., 2022; Heeringa & Berglund, 2020; Owens et al., 2021). Covariates included the abovementioned variables and total intracranial volume. The statistical threshold was set at *P* < 0.05, false discovery rate (FDR)-corrected using the Benjamini–Hochberg method. Thereafter, corrections for the family-wise error (FWE; *P* < 0.05) rate were performed using the Bonferroni method for multiple group comparisons.

**Supplementary Results 1. Additional demographics**

Demographic characteristics of each ADHD subtype are shown in **Supplementary Table 2**. The ADHD-C type had significantly higher scores for ADHD, conduct disorder, and oppositional defiant disorder symptoms than the ADHD-A (*P*s < 0.001 for ADHD and conduct scores; *P* = 0.002 for oppositional defiant score) and ADHD-B types (*P* = 0.016 for ADHD score; *P* < 0.001 for conduct score; *P* = 0.002 for oppositional defiant score), although each ADHD subtype had significantly higher scores for each clinical symptom than the non-ADHD group (*P*s < 0.001).

**Supplementary Results 2. Cognitive functional characteristics adjusted for comorbidities**

As shown in **Supplementary Table 3** and **Supplementary Figure 2**, the main effect of group in the linear mixed-effects model showed that the ADHD-A type had significantly higher levels of cognitive control, processing speed, working memory, episodic memory, and language than the non-ADHD group (FDR, *Ps* < 0.001; FWE, *Ps* < 0.001). Moreover, the main effect of group showed that the ADHD-B type had significantly lower levels of cognitive control and processing speed than the non-ADHD group (FDR, *Ps* < 0.001; FWE, *Ps* < 0.001). As for weak results, the main effect of group showed that the ADHD-B type had a lower level of episodic memory than the non-ADHD group (FDR, *P* = 0.026; FWE, *P* = 0.078). Furthermore, the main effect of group showed that the ADHD-C type had lower levels of cognitive control, working memory, episodic memory, and language than the non-ADHD group (FDR, *P* < 0.001; FWE, *P*s < 0.001).

**Supplementary Results 3. Brain structural characteristics adjusted for comorbidities**

As shown in **Supplementary Table 4** and **Supplementary Figure 3**, the main effect of group in the linear mixed-effects model showed that the ADHD-C type displayed a smaller volume of the right lateral orbitofrontal cortex (FDR, *P* = 0.011; FWE, *P* = 0.033), whereas the ADHD-A and ADHD-B types did not show such significant differences in regional brain volumes.

**Supplementary Discussion.**

For additional demographics in ADHD subtypes (**Supplementary Table 2**), the Child Behavior Checklist (CBCL) data revealed higher symptoms of ADHD, conduct disorder, and oppositional defiant disorder in the ADHD-C type than those in the ADHD-A, ADHD-B, and non-ADHD groups. In addition, the ADHD-A and ADHD-B types had higher symptoms of ADHD, conduct disorder, and oppositional defiant disorder than the non-ADHD group. Notably, the ADHD-C type displayed more severe ADHD symptoms and psychiatric problems of conduct and oppositional defiant disorders than the other subtypes. As each executive function has a differential predictive value for ADHD symptoms (Cai et al., 2023; Mohamed et al., 2021; Sabhlok et al., 2022; Salari, Bohlin, Rydell, & Thorell, 2017), this study also demonstrated distinct clinical symptom characteristics for each subtype, classified by cognitive function domains. These aspects of cognitive functioning heterogeneity could contribute to the identification of distinct clinical features among ADHD subtypes, highlighting the need for different approaches and considerations for the diagnosis and treatment of ADHD across these subtypes.

Based on the results of the linear mixed-effects model adjusted for these comorbidities, compared with the non-ADHD group, the ADHD-A type was characterised by high cognitive function, the ADHD-B type was characterised by low cognitive control and processing speed, and the ADHD-C type was characterised by strikingly low cognitive control, working memory, episodic memory, and language performances (**Supplementary Table 3** and **Supplementary Figure 2**). These findings suggest that individuals with ADHD-C may exhibit vulnerabilities in cognitive control, working memory, episodic memory, and language functions, regardless of the presence of comorbidities. In contrast, individuals with ADHD-A demonstrated superiority in all cognitive functions measured by the NIH Toolbox, and individuals with ADHD-B showed more pronounced vulnerabilities in processing speed and cognitive control.

Moreover, the ADHD-C type was characterized by volume reductions of the right lateral orbitofrontal cortex compared with the non-ADHD group (**Supplementary Table 4** and **Supplementary Figure 3**). By contrast, the ADHD-A and ADHD-B types did not show brain structural changes compared with the non-ADHD group. These findings persisted even when controlling for the presence of comorbidities, presumably because the right lateral orbitofrontal cortex region is essential for the ADHD-C symptomatology. This may provide valuable insights into the specific structural anomalies of this brain region in the ADHD-C type.

**Supplementary References.**

Bernanke, J., Luna, A., Chang, L., Bruno, E., Dworkin, J., & Posner, J. (2022). Structural brain measures among children with and without ADHD in the Adolescent Brain and Cognitive Development Study cohort: a cross-sectional US population-based study. *The Lancet. Psychiatry*, *9*(3), 222–231. doi:10.1016/S2215-0366(21)00505-8.

Bishop, C., Mulraney, M., Rinehart, N., & Sciberras, E. (2019). An examination of the association between anxiety and social functioning in youth with ADHD: A systematic review. *Psychiatry Research*, *273*, 402–421. doi:10.1016/j.psychres.2019.01.039.

Cai, W., Warren, S. L., Duberg, K., Yu, A., Hinshaw, S. P., & Menon, V. (2023). Both reactive and proactive control are deficient in children with ADHD and predictive of clinical symptoms. *Translational Psychiatry*, *13*(1), 179. doi:10.1038/s41398-023-02471-w.

Hamatani, S., Hiraoka, D., Makita, K., Tomoda, A., & Mizuno, Y. (2022). Longitudinal impact of COVID-19 pandemic on mental health of children in the ABCD study cohort. *Scientific Reports*, *12*(1), 19601. doi:10.1038/s41598-022-22694-z.

Heeringa, S., & Berglund, P. A. (2020). A guide for population-based analysis of the adolescent brain cognitive development (ABCD) study baseline data. Preprint at <https://doi.org/10.1101/2020.02.10.942011>.

Hiraoka, D., Makita, K., Hamatani, S., Tomoda, A., & Mizuno, Y. (2023). Effects of prenatal cannabis exposure on developmental trajectory of cognitive ability and brain volumes in the adolescent brain cognitive development (ABCD) study. *Developmental Cognitive Neuroscience*, *60*, 101209. doi:10.1016/j.dcn.2023.101209.

Mohamed, S. M. H., Butzbach, M., Fuermaier, A. B. M., Weisbrod, M., Aschenbrenner, S., Tucha, L., & Tucha, O. (2021). Basic and complex cognitive functions in Adult ADHD. *PLoS One*, *16*(9), e0256228. doi:10.1371/journal.pone.0256228.

Owens, M. M., Allgaier, N., Hahn, S., Yuan, D., Albaugh, M., Adise, S., … Garavan, H. (2021). Multimethod investigation of the neurobiological basis of ADHD symptomatology in children aged 9-10: baseline data from the ABCD study. *Translational Psychiatry*, *11*(1), 64. doi:10.1038/s41398-020-01192-8.

Sabhlok, A., Malanchini, M., Engelhardt, L. E., Madole, J., Tucker-Drob, E. M., & Harden, K. P. (2022). The relationship between executive function, processing speed, and attention-deficit hyperactivity disorder in middle childhood. *Developmental Science*, *25*(2), e13168. doi:10.1111/desc.13168.

Salari, R., Bohlin, G., Rydell, A. M., & Thorell, L. B. (2017). Neuropsychological functioning and attachment representations in early school age as predictors of ADHD symptoms in late adolescence. *Child Psychiatry and Human Development*, *48*(3), 370–384. doi:10.1007/s10578-016-0664-1.

Seo, J. C., Jon, D. I., Shim, S. H., Sung, H. M., Woo, Y. S., Hong, J., … Bahk, W. M. (2022). Prevalence and comorbidities of attention deficit hyperactivity disorder among adults and children/adolescents in Korea. *Clinical Psychopharmacology and Neuroscience: The Official Scientific Journal of the Korean College of Neuropsychopharmacology*, *20*(1), 126–134. doi:10.9758/cpn.2022.20.1.126.

Tistarelli, N., Fagnani, C., Troianiello, M., Stazi, M. A., & Adriani, W. (2020). The nature and nurture of ADHD and its comorbidities: A narrative review on twin studies. *Neuroscience and Biobehavioral Reviews*, *109*, 63–77. doi:10.1016/j.neubiorev.2019.12.017.

Wu, M., Joubran, E., Kumar, D., Assadi, N. D., & Nguyen, H. (2023). Variations in anxiety and related psychiatric comorbidity levels among youths with individual diagnoses of autism spectrum disorder or attention deficit hyperactivity disorder and those with both diagnoses. *Cureus*, *15*(7), e41759. doi:10.7759/cureus.41759.

**Supplementary Tables**

Supplementary Table 1. Variables used in this study

| Variable | Data file |
| --- | --- |
| NIH Toolbox: flanker inhibitory control and attention | abcd_tbss01 |
| NIH Toolbox: dimensional change card sort |  |
| NIH Toolbox: pattern comparison processing speed |  |
| NIH Toolbox: list sorting working memory |  |
| NIH Toolbox: picture sequence memory |  |
| NIH Toolbox: picture vocabulary |  |
| NIH Toolbox: oral reading recognition |  |
| Grey matter volumes for 34 cortical regions (Desikan atlas-based classification; 68 regions in total) | abcd_smrip101 |
| Grey matter volumes for 6 subcortical regions (atlas-based classification; 12 regions in total), intracranial volume | abcd_smrip201 |
| Handedness | abcd_ehis01 |
| K-SADS | abcd_ksad01 |
| FreeSurfer quality control | abcd_imgincl01 |
| Site ID | abcd_lt01 |
| MRI scanner number | abcd_mri01 |
| Medication use | medsy01 |
| Race/ethnicity, sibling status, twin or triplet status | acspsw03 |
| Home environment (education and income) | pdem02 |
| CBCL | abcd_cbcls01 |
| Pubertal status | abcd_ppdms01 (parent/guardian)  abcd_ypdms01 (participants) |

CBCL, Child Behavior Checklist; K-SADS, Kiddie Schedule for Affective Disorders and Schizophrenia; MRI, magnetic resonance imaging.

Supplementary Table 2. Demographics of each ADHD subtype and the non-ADHD group

| Characteristics | ADHD-A  (n = 212) | ADHD-B  (n = 190) | ADHD-C  (n = 254) | Non-ADHD  (n = 6 601) | *P*-value |
| --- | --- | --- | --- | --- | --- |
| Age (months) | 119.48 (7.97) | 118.87 (7.37) | 118.02 (7.23) | 119.04 (7.48) | *NA* |
| Parental education (years) | 15.98 (2.33) | 15.79 (2.38) | 14.52 (2.33) | 15.22 (2.62) | ADHD-A vs ADHD-B: > 0.99  ADHD-A vs ADHD-C: < 0.001  ADHD-B vs ADHD-C: < 0.001  ADHD-A vs non-ADHD: < 0.001  ADHD-B vs non-ADHD: 0.025  ADHD-C vs non-ADHD: < 0.001 |
| Pubertal status (score) | 1.53 (0.44) | 1.47 (0.44) | 1.66 (0.57) | 1.61 (0.50) | ADHD-A vs ADHD-B: > 0.99  ADHD-A vs ADHD-C: 0.0317  ADHD-B vs ADHD-C: < 0.001  ADHD-A vs non-ADHD: 0.116  ADHD-B vs non-ADHD: < 0.001  ADHD-C vs non-ADHD: 0.791 |
| Sex (n)  Male  Female | 136 (64.15)  76 (35.85) | 143 (75.26)  47 (24.74) | 172 (67.72)  82 (32.28) | 3 328 (50.42)  3 273 (49.58) | ADHD-A vs ADHD-B: 0.127  ADHD-A vs ADHD-C: > 0.99  ADHD-B vs ADHD-C: 0.622  ADHD-A vs non-ADHD: < 0.001  ADHD-B vs non-ADHD: < 0.001  ADHD-C vs non-ADHD: < 0.001 |
| Race/ethnicity (n) | | | | | |
| White | 140 (66.03) | 110 (57.89) | 102 (40.16) | 3 477 (52.67) | ADHD-A vs ADHD-B: 0.688  ADHD-A vs ADHD-C: < 0.001  ADHD-B vs ADHD-C: 0.00186  ADHD-A vs non-ADHD: 0.001  ADHD-B vs non-ADHD: > 0.99  ADHD-C vs non-ADHD: < 0.001 |
| Black | 15 (7.08) | 29 (15.26) | 66 (25.98) | 957 (14.50) | ADHD-A vs ADHD-B: 0.082  ADHD-A vs ADHD-C: < 0.001  ADHD-B vs ADHD-C: 0.055  ADHD-A vs non-ADHD: 0.02  ADHD-B vs non-ADHD: > 0.99  ADHD-C vs non-ADHD: < 0.001 |
| Hispanic | 33 (15.57) | 22 (11.58) | 43 (16.93) | 1 345 (20.37) | ADHD-A vs ADHD-B: > 0.99  ADHD-A vs ADHD-C: > 0.99  ADHD-B vs ADHD-C: 0.895  ADHD-A vs non-ADHD: 0.618  ADHD-B vs non-ADHD: 0.023  ADHD-C vs non-ADHD: > 0.99 |
| Asian | 5 (2.36) | 0 (0) | 0 (0) | 144 (2.18) | ADHD-A vs ADHD-B: 0.470  ADHD-A vs ADHD-C: 0.220  ADHD-B vs ADHD-C: *NA*  ADHD-A vs non-ADHD: > 0.99  ADHD-B vs non-ADHD: 0.36  ADHD-C vs non-ADHD: 0.16 |
| Other | 19 (8.96) | 29 (15.26) | 43 (16.93) | 677 (10.26) | ADHD-A vs ADHD-B: 0.440  ADHD-A vs ADHD-C: 0.103  ADHD-B vs ADHD-C: > 0.99  ADHD-A vs non-ADHD: > 0.99  ADHD-B vs non-ADHD: 0.210  ADHD-C vs non-ADHD: 0.005 |
| Annual household income (US$) (n) | | | | | |
| < 49 999  50 000–74 999  75 000–99 999  100 000–199 999  ≥ 200 000 | 31 (14.62)  33 (15.67)  36 (16.98)  71 (33.49)  30 (14.15) | 56 (29.47)  18 (9.47)  34 (17.89)  54 (28.42)  18 (9.47) | 102 (40.16)  34 (13.39)  26 (10.24)  42 (16.54)  18 (7.09) | 1 735 (26.28)  822 (12.45)  917 (13.89)  1 907 (28.89)  680 (10.30) | ADHD-A vs ADHD-B: 0.035  ADHD-A vs ADHD-C: < 0.001  ADHD-B vs ADHD-C: 0.003  ADHD-A vs non-ADHD: 0.004  ADHD-B vs non-ADHD: > 0.99  ADHD-C vs non-ADHD: < 0.001 |
| CBCL (score) | | | | | |
| ADHD symptom | 62.19 (7.79) | 63.27 (7.54) | 64.58 (7.38) | 52.10 (4.10) | ADHD-A vs ADHD-B: 0.100  ADHD-A vs ADHD-C: < 0.001  ADHD-B vs ADHD-C: 0.016  ADHD-A vs non-ADHD: < 0.001  ADHD-B vs non-ADHD: < 0.001  ADHD-C vs non-ADHD: < 0.001 |
| Depressive symptom | 58.32 (7.67) | 59.61 (8.15) | 59.57 (7.80) | 52.95 (4.99) | ADHD-A vs ADHD-B: 0.090  ADHD-A vs ADHD-C: 0.068  ADHD-B vs ADHD-C: > 0.99  ADHD-A vs non-ADHD: < 0.001  ADHD-B vs non-ADHD: < 0.001  ADHD-C vs non-ADHD: < 0.001 |
| Symptom of anxiety disorder | 57.62 (8.44) | 58.80 (9.26) | 59.59 (10.37) | 52.96 (5.51) | ADHD-A vs ADHD-B: 0.290  ADHD-A vs ADHD-C: 0.002  ADHD-B vs ADHD-C: 0.992  ADHD-A vs non-ADHD: < 0.001  ADHD-B vs non-ADHD: < 0.001  ADHD-C vs non-ADHD: < 0.001 |
| Symptom of conduct disorder | 56.72 (7.72) | 57.50 (8.03) | 59.69 (8.82) | 52.36 (4.67) | ADHD-A vs ADHD-B: 0.750  ADHD-A vs ADHD-C: < 0.001  ADHD-B vs ADHD-C: < 0.001  ADHD-A vs non-ADHD: < 0.001  ADHD-B vs non-ADHD: < 0.001  ADHD-C vs non-ADHD: < 0.001 |
| Symptom of oppositional defiant disorder | 58.85 (8.17) | 58.51 (8.11) | 60.48 (8.87) | 52.75 (4.42) | ADHD-A vs ADHD-B: > 0.99  ADHD-A vs ADHD-C: 0.002  ADHD-B vs ADHD-C: 0.002  ADHD-A vs non-ADHD: < 0.001  ADHD-B vs non-ADHD: < 0.001  ADHD-C vs non-ADHD: < 0.001 |
| Symptom of somatic disorder | 57.76 (7.47) | 57.84 (7.16) | 58.25 (7.86) | 55.25 (6.41) | ADHD-A vs ADHD-B: > 0.99  ADHD-A vs ADHD-C: > 0.99  ADHD-B vs ADHD-C: > 0.99  ADHD-A vs non-ADHD: < 0.001  ADHD-B vs non-ADHD: < 0.001  ADHD-C vs non-ADHD: < 0.001 |

Data are presented as the mean (*SD*) or n (%). *P*-values for age, education, puberty, income, ADHD, depressive symptoms, and CBCL scores are from analyses of variance for group differences. *P*-values for sex ratio and race/ethnicity ratio were obtained from chi-square tests for group differences. ADHD, attention-deficit/hyperactivity disorder; CBCL, Child Behavior Checklist; *NA*, not applicable; *SD*, standard deviation.

**Supplementary Table 3**. Differences in behavioural results between each ADHD subtype and the non-ADHD group adjusted for comorbidities

| Variable | *b* | 95% CI | *β* | 95% CI | *R*^2^ | *t* | *F* | *d.f.* | FDR-*P* | FWE-*P* |
| --- | --- | --- | --- | --- | --- | --- | --- | --- | --- | --- |
| ADHD-A  CC  PS  WM  EM  LF | 4.40  8.46  6.60  10.36  4.93 | 2.03, 6.77  4.23, 12.70  3.94, 9.26  7.36, 13.37  2.28, 7.58 | 0.06  0.07  0.08  0.12  0.06 | 0.03, 0.10  0.03, 0.10  0.05, 0.12  0.08, 0.15  0.03, 0.09 | 0.05  0.05  0.10  0.08  0.20 | 3.64  3.92  4.87  6.76  3.65 | 13.23  15.34  23.69  45.71  13.30 | 3 146.74  3 147.49  3 154.98  3 146.63  3 112.37 | < 0.001  < 0.001  < 0.001  < 0.001  < 0.001 | < 0.001  < 0.001  < 0.001  < 0.001  < 0.001 |
| ADHD-B  CC  PS  WM  EM  LF | -3.46  -7.98  -1.34  -2.10  0.26 | -4.81, -2.11  -10.40, -5.55  -2.86, 0.18  -3.80, -0.40  -1.24, 1.76 | -0.09  -0.12  -0.03  -0.04  0.006 | -0.13, -0.06  -0.15, -0.08  -0.07, 0.004  -0.08, -0.008  -0.03, 0.04 | 0.05  0.06  0.09  0.07  0.20 | 5.03  6.45  1.73  2.42  0.34 | 25.33  41.64  3.00  5.85  0.11 | 3 129.67  3 130.07  3 134.07  3 115.95  3 069.55 | < 0.001  < 0.001  0.104  0.026  0.737 | < 0.001  < 0.001  0.312  0.078  > 0.999 |
| ADHD-C  CC  PS  WM  EM  LF | -2.11  -0.86  -5.31  -2.78  -3.24 | -2.97, -1.24  -2.42, 0.69  -6.28, -4.35  -3.86, -1.69  -4.19, -2.29 | -0.09  -0.02  -0.19  -0.09  -0.11 | -0.12, -0.05  -0.06, 0.02  -0.22, -0.15  -0.13, -0.05  -0.14, -0.08 | 0.05  0.04  0.13  0.08  0.21 | 4.77  1.09  10.82  5.02  6.68 | 22.77  1.19  117.02  25.22  44.60 | 3 127.94  3 123.33  3 134.83  3 118.74  3 091.14 | < 0.001  0.276  < 0.001  < 0.001  < 0.001 | < 0.001  0.828  < 0.001  < 0.001  < 0.001 |

ADHD, attention-deficit/hyperactivity disorder; *b*, unstandardized coefficient; *β*, standardized coefficient; CC, cognitive control; CI, confidence interval; *d.f*., degree of freedom; EM, episodic memory; FDR, false discovery rate; FWE, family-wise error; LF, language function; PS, processing speed; WM, working memory.

**Supplementary Table 4.** Brain areas with significantly smaller volumes in the ADHD-C group than in the non-ADHD group adjusted for comorbidities

| Brain | *b* | 95% CI | *β* | 95% CI | *R*^2^ | *t* | *F* | *d.f.* | FDR-*P* | FWE-*P* |
| --- | --- | --- | --- | --- | --- | --- | --- | --- | --- | --- |
| R-LOFC | -106.59 | -161.40, -51.77 | -0.05 | -0.07, -0.02 | 0.38 | 3.81 | 14.53 | 3 075.52 | 0.011 | 0.034 |

ADHD, attention-deficit/hyperactivity disorder; *b*, unstandardized coefficient; *β*, standardized coefficient; CI, confidence interval; *d.f*., degree of freedom; FDR, false discovery rate; FWE, family-wise error; R-LOFC, right lateral orbitofrontal cortex.

**Supplementary Figures**


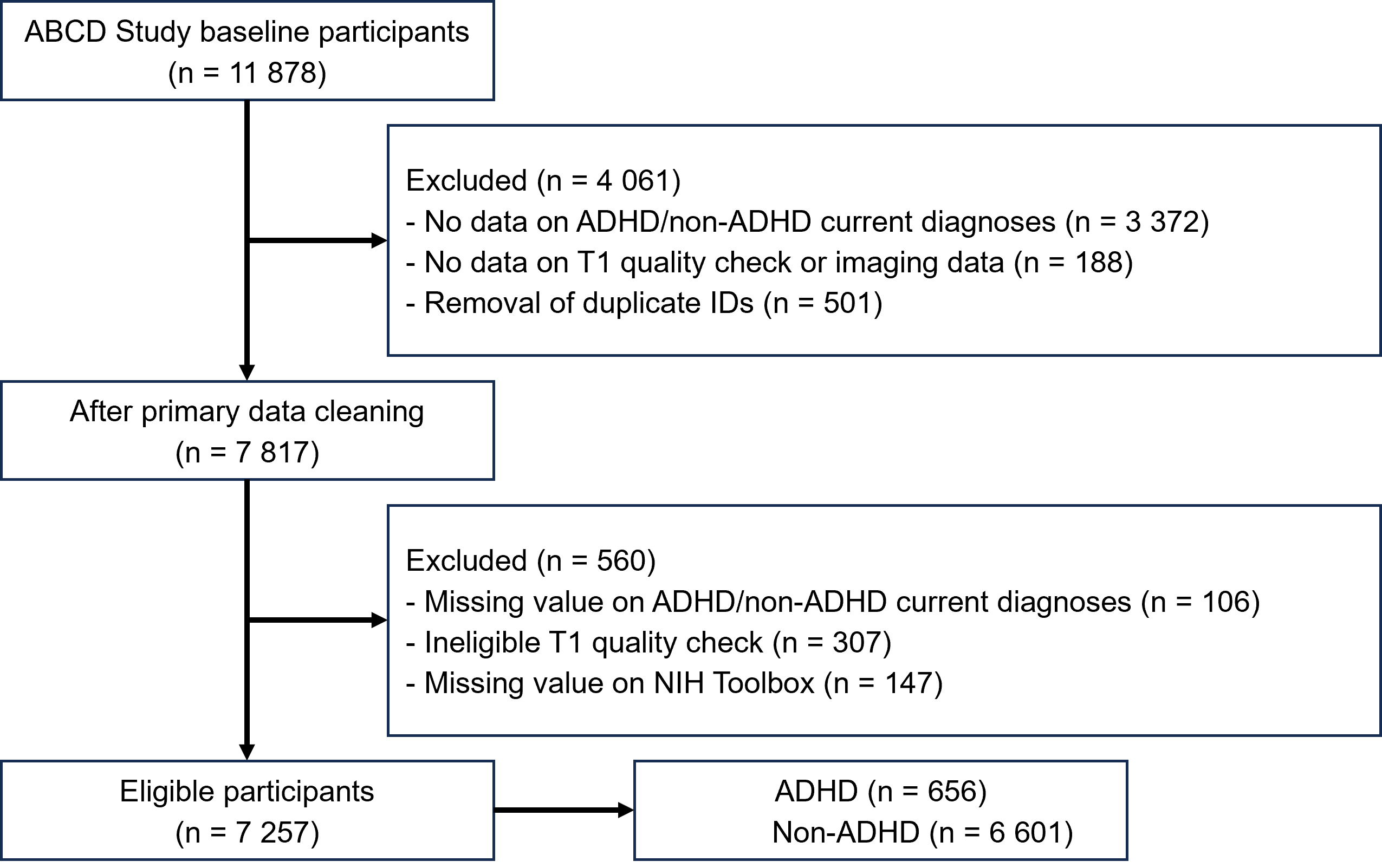


Supplementary Figure 1. Flowchart of the sampling procedure. ABCD, Adolescent Brain Cognitive Development; ADHD, attention-deficit/hyperactivity disorder.


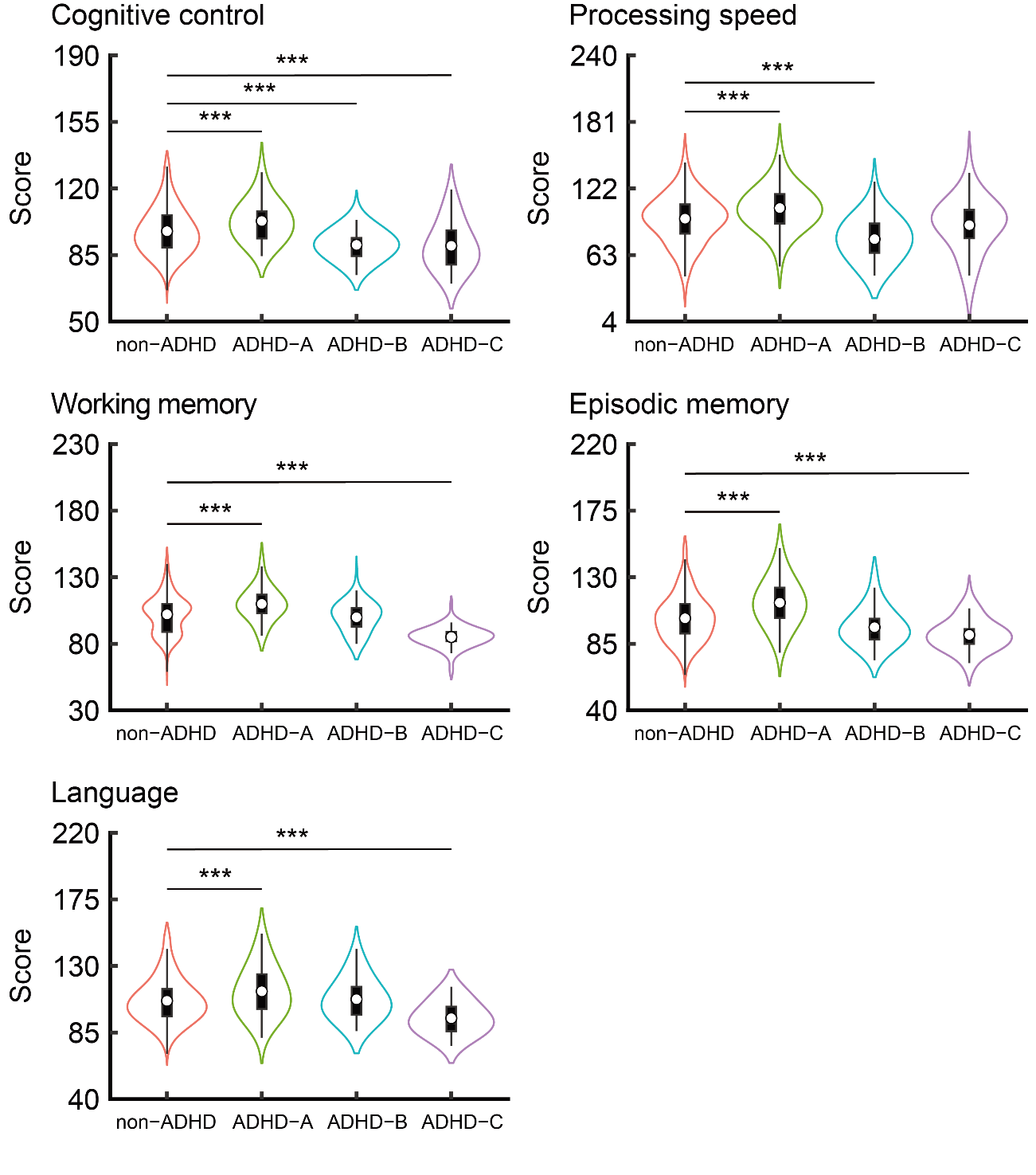


Supplementary Figure 2. Cognitive functional characteristics adjusted for comorbidities by ADHD subtype. Based on FDR- and FWE-corrected thresholds (*P* < 0.05), the ADHD-A group shows in all cognitive functions better performances than the non-ADHD group. The ADHD-B group shows poorer performances in cognitive control and processing speed than the non-ADHD group. The ADHD-C group shows poorer performances in cognitive control, working memory, episodic memory, and language than the non-ADHD group. Data are shown as the mean (*SD*). *** FWE-*P* < 0.001. ADHD, attention-deficit/hyperactivity disorder; FDR, false discovery rate; FWE, family-wise error; *SD*, standard deviation.


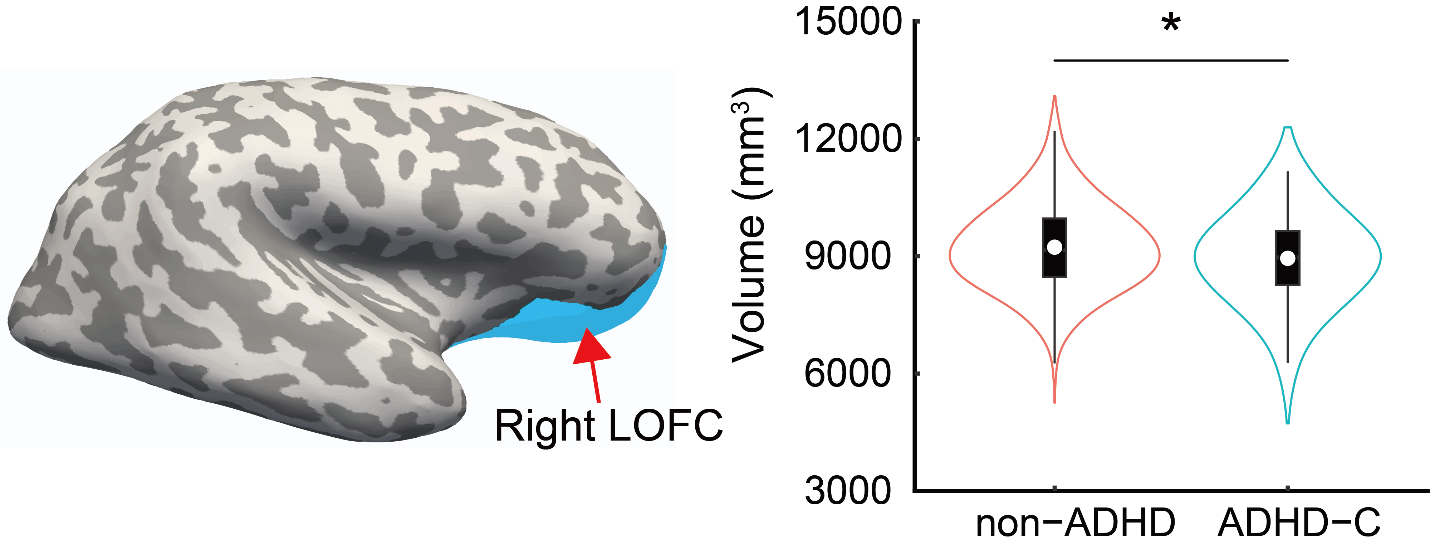


Supplementary Figure 3. Brain structural characteristics adjusted for comorbidities in the ADHD-C type. Based on FDR- and FWE-corrected thresholds (*P* < 0.05), the ADHD-C type has a smaller volume of the right lateral orbitofrontal cortex than the non-ADHD group. Data are shown as the mean (*SD*). * FWE-*P* < 0.05. ADHD, attention-deficit/hyperactivity disorder; FDR, false discovery rate; FWE, family-wise error; LOFC, lateral orbitofrontal cortex; *SD*, standard deviation.
